# Sustainable implementation efforts in physio- and occupational therapy: a scoping review

**DOI:** 10.1186/s43058-024-00676-8

**Published:** 2024-12-12

**Authors:** Johanna Fritz, Sara Landerdahl Stridsberg, Riikka Holopainen

**Affiliations:** 1 School of Health, Care and Social Welfare, Mälardalen University, Box 883, Västerås, SE-721 23 Sweden; 2 Mälardalen University, University Library, Västerås, Sweden; 3 Faculty of Sports and Health Sciences, University of Jyväskylä, Jyväskylä, Finland; 4 Southern-Savo Healthcare District, Mikkeli, Finland

**Keywords:** Behavior change techniques, Clinical behavior, Evidence-based practice, Implementation, Sustainability, Implementation strategy, Maintenance, Occupational therapist, Physiotherapist

## Abstract

**Background:**

Health care professionals often fail to adhere to evidence-based guidelines. The implementation of evidence-based methods in health care requires systematic support, but it is still unclear which strategies support professional adherence to clinical practice guidelines. Behavior change techniques can contribute to a more detailed description of implementation strategies. The aim of this scoping review was to explore the nature of studies investigating the sustainability of physiotherapists’ (PTs’) and occupational therapists’ (OTs’) clinical behavior when implementing evidence-based methods in health care. Two research questions were addressed: (1) Which implementation strategies are used in studies that have experienced sustained and unsustained changes in the clinical behavior of PTs and OTs? (2) Which behavior change techniques are used in studies involving sustained and unsustained changes in the clinical behavior of PTs and OTs?

**Methods:**

The scoping review was carried out in accordance with recommendations and the PRISMA-ScR checklist. Six databases were searched. Studies evaluating changes in the clinical behavior of PTs or OTs before and at least 6 months after the end of an implementation intervention were included.

**Results:**

A total of 5130 studies were screened, and 29 studies were included. Twenty-one studies reported sustained results, and 8 studies reported unsustained results. The studies reporting sustained clinical behavior used in median 7 implementation strategies, 45% used a 12–24-month implementation support period, and 86% of the interventions were theory-based. Twenty-two implementation strategies were identified among the included studies. Only two of these defined the implementation strategies in terms of behavior change techniques.

**Conclusions:**

Studies reporting sustained results were characterized by the use of longer implementation periods, more implementation strategies, more theory-based interventions, and more behavior change techniques. Audit and feedback, resources, problem solving, and communities of practice were implementation strategies, and problem solving, demonstration of behavior, and social support were behavior change techniques that were more common in studies with sustained results of PTs’ and OTs’ clinical behavior. Our study also highlights the importance of well-described implementation studies.

**Registration:**

The protocol for the scoping review has been registered in the Open Science Framework, OSF registry (10.17605/OSF.IO/DUYQM).

**Supplementary Information:**

The online version contains supplementary material available at 10.1186/s43058-024-00676-8.

Contributions to the literature

- This scoping review contributes to fill the research gap on sustainable results when implementing evidence-based methods in health care.
- The findings of this review describe characteristics of implementation strategies resulting in sustained changes of physiotherapists’ and occupational therapists’ clinical behavior.
- This study compares strategies resulting in sustained and unsustained clinical behavior change.
- This scoping review highlight the importance of using valid methods when evaluating professionals’ clinical behavior change.

## Background

Health care professionals often fail to adhere to evidence-based guidelines and physiotherapists and occupational therapists are no exceptions. For example, in their review, Zadro et al. reported that for musculoskeletal conditions, 54% of physiotherapists chose recommended treatments, and 43% used treatments that were not recommended [1]. The implementation of new evidence-based methods means that health care professionals need to change their clinical behavior, which remains a challenge despite more than two decades of research [2]. Ataman et al. [3] reported that only 54% of evidence-based practices in rehabilitation are sustained. Similar findings regarding professionals’ adherence to clinical practice guidelines decreasing more than one year after implementation in approximately half of the cases were reported by Ament et al. [4]. Sustainability planning is rarely reported in implementation studies, but if authors have reported sustainability planning, the practice was sustained 94% of the time [3].

Implementation is defined as a systematic process for promoting the uptake of evidence-based methods to improve quality of care, as seen from the perspective of the individual and the organization [5, 6]. Sustainability is commonly defined as a professional’s adherence to the implemented clinical guidelines [3, 4]. In contrast to sustained uptake, implementation science typically focuses on the early use of evidence-based methods. More than ten years ago, Wiltsey Stirman et al. [7] recommended considering sustainability in implementation research, and the recommendation is still just as relevant. Among 400 studies about implementation outcomes, only 15.1% examined sustainability [8]. Among studies focusing on the sustainability of implementation efforts, there is variability in the methods and outcomes used [7, 8]. According to the Reach, Effectiveness, Adoption, Implementation and Maintenance (RE-AIM) framework, the specific time frame for assessing sustainability varies from relatively short-term intervals—6 months after the end of the implementation support period—to 12 months or longer [6].

The implementation of evidence-based methods in health care usually requires systematic support, which refers to one or several strategies used to support implementation [9] The Cochrane Effective Practice and Organization of Care (EPOC) group taxonomy [10] can be used to standardize the naming and description of these strategies. Education seems only to change professionals’ knowledge and attitudes and to bring temporary changes to their way of working [11, 12]. There is little evidence concerning the relationship between implementation support strategies and implementation outcomes [8]. However, it appears that some strategies support the initiation of the use of new methods and that other strategies support the sustained use of these methods [13]. Psychological behavior change theories can help in understanding and explaining what influences implementation outcomes [14]. To make psychological theories for behavior change easier to apply in implementation, Michie et al. developed the COM-B system based on 19 frameworks of behavior change [15]. According to the COM-B system, a behavior is dependent on an interacting system of essential conditions involving capability, opportunity, and motivation and interventions targeting these conditions. The most effective interventions for both initiating and sustaining behavioral change are those targeting all these conditions [15]. Implementation strategies also need to support sustainable clinical behavior change. Ataman et al. [3] reported that contextual circumstances are important for sustainability, but it is still unclear which strategies support professionals’ long-term adherence to clinical practice guidelines.

Many implementation studies have used only attitudes and knowledge as outcomes when exploring the effectiveness of implementation strategies [16–24]. However, to be able to evaluate the professional’s adherence to clinical guidelines, the outcomes need to target the professional’s clinical behavior. Furthermore, there is often a discrepancy between reported and observed clinical behavior. For example, Fritz et al. [25] found that even though physiotherapists perceived that they practiced a behavioral medicine approach, often the intended approach was not observable in video-recorded appointments. Generally, implementation strategies are poorly reported in studies and lack a detailed description, which makes them difficult to compare. The descriptions that exist need consistent terminology [26, 27]. Classifying implementation support strategies according to a behavior change technique (i.e., a strategy that support an individual to change their behavior) taxonomy is recommended to offer a more detailed description of the implementation strategies and a common language for reporting these strategies [28]. Crawshaw et al. found that the implementation strategy “audit and feedback” consisted of a median of five (range 1–29) behavior change techniques [29]. Thus, behavior change techniques can be a tool for a more detailed description of an implementation strategy.

The aim of this scoping review was to explore the nature of studies investigating the sustainability of physiotherapists’ (PTs’) and occupational therapists’ (OTs’) clinical behavior when implementing evidence-based methods in health care.

This review addressed two research questions:

- Which implementation strategies are used in studies reporting sustained and unsustained changes in the clinical behavior of PTs and OTs?
- Which behavior change techniques are used in studies reporting sustained and unsustained changes in the clinical behavior of PTs and OTs?

## Methods

The scoping review was carried out based on recommendations when little is known within the area of interest [30, 31] and stage 1–5 in the methodological framework described by Levac et al. [32]. The Preferred Reporting Items for Systematic Reviews and Meta-Analyses statement for scoping reviews (PRISMA-ScR) checklist [31] was used to report the results (see Additional file 1). The protocol for the scoping review has been registered in the Open Science Framework, OSF registry (10.17605/OSF.IO/DUYQM) [33].

### Search strategy and data sources

With the assistance of a librarian (SLS), we conducted a comprehensive search of PubMed (pubmed.ncbi.nlm.nih.gov), Cinahl Plus (EBSCOhost), Scopus (scopus.com), Cochrane CENTRAL (cochranelibrary.com/central), APA PsycInfo (EBSCOhost) and PEDro (https://pedro.org.au) on January 12, 2023, and an additional search for new studies was conducted in October 2023. To make the search strategy correspond to the aim of the review, the population, intervention, outcome (PIO) framework was used to define the search/search blocks (see Table 1). The free text search terms are listed in Table 1, and a detailed overview of the search terms, both free text and controlled vocabulary, for each database is presented in Additional file 2. The authors collaboratively determined the search strategy and selected the databases based on the rationale of the study and the search strategy used by Ataman et al. [3]. The search strategy was developed iteratively and tested before the final search was conducted. The number of search terms was selected to maximize the search capability for this review. In addition, the studies included in the review by Ataman [3] and the reference lists of the included full-text studies were examined to identify other potentially relevant studies. Due to the limited search function in the PEDro database, the search in that database was greatly simplified. When possible, the search was limited to the English language.

**Table 1:** Search terms

PIO concept	Search terms
**Population**	“physical therapist*” OR physiotherapist* OR “occupational therapist*”
**Intervention**	implement* OR uptake OR diffusion OR disseminat* OR adopt* OR “knowledge transl*” OR “continuing education” OR evidence-informed OR evidence-based OR “evidence based”
**Outcome**	“implementation outcome*” OR institutionalization OR normalization OR normalization OR re-invention OR “continued use” OR assimilation OR “long term use” OR “use long term” OR “program continuation” OR “implementation continuation” OR follow-up OR evaluat* OR before-after OR before-and-after OR maintain* OR sustain* OR durability OR routinization OR routinization OR continuation OR “Policy Compliance” OR “Protocol Compliance” OR “Institutional Adherence” OR “Guideline Adherence”

### The eligibility criteria

We included quantitative studies or studies with mixed method designs that evaluated the changes in the clinical behavior of PTs or OTs after an implementation intervention. The studies had to include pre- and follow-up measurements after at least 6 months to be considered to evaluate sustainability. Results that were reported as a behavior change after at least 6 months were considered as sustained results. A description of the implementation strategies used had to be presented. We included only peer-reviewed articles written in English. Only qualitative studies, study protocols, literature reviews, and conference abstracts, as well as studies focusing on undergraduate PT/OT students or studies evaluating only professionals’ knowledge and attitude, were excluded. Qualitative studies were excluded because of difficulties in objectively determining whether a change in clinical behavior existed.

### Data collection and extraction

The web-based systematic review software platform Covidence (Veritas Health Innovation, Melbourne, Australia. Available at www.covidence.org.) was used for the deduplication, screening, and data extraction processes. All titles and abstracts were screened by two of the authors (JF, RH) independently. If the decision for inclusion was uncertain, the study was included in the next step. All full texts were screened by the same two reviewers independently. Discrepancies at both levels of screening were discussed and resolved collaboratively. One of the reviewers was an author of one of the identified studies. The screening process with two separate reviewers reduced the risk of bias. Data from the included full-text articles were extracted by both reviewers independently. The following data were collected: title, authors, year, country, aim, design, participants, setting, intervention to be implemented, implementation support strategy, sustainability strategy, theoretical basis for the implementation support strategy, implementation support period, time point for follow-up, and results of the outcomes regarding clinical behavior. The extracted data were exported from Covidence into an Excel spreadsheet and from there to multiple matrices (Additional files 3–4) to make the data manageable for synthesis and reporting.

### Data synthesis and analysis

We conducted a descriptive analysis of the data from all included studies to answer the research questions. Characteristics, implementation support strategies and behavior change techniques were calculated and reported as frequencies, averages, and percentages. The studies reporting sustained results were compared with the studies reporting unsustained results. Data regarding implementation strategies were grouped and named in accordance with the EPOC group taxonomy [10] and implementation support strategies were coded in accordance with Michie et al.’s taxonomy for behavior change techniques [34]. The most commonly used implementation strategies (i.e., implementation strategies used in at least half of the studies) were categorized according to the COM-B system [15]. All analysis were performed by two of the authors (JF, RH). Discrepancies were discussed and resolved collaboratively.

## Results

**Fig. 1:**
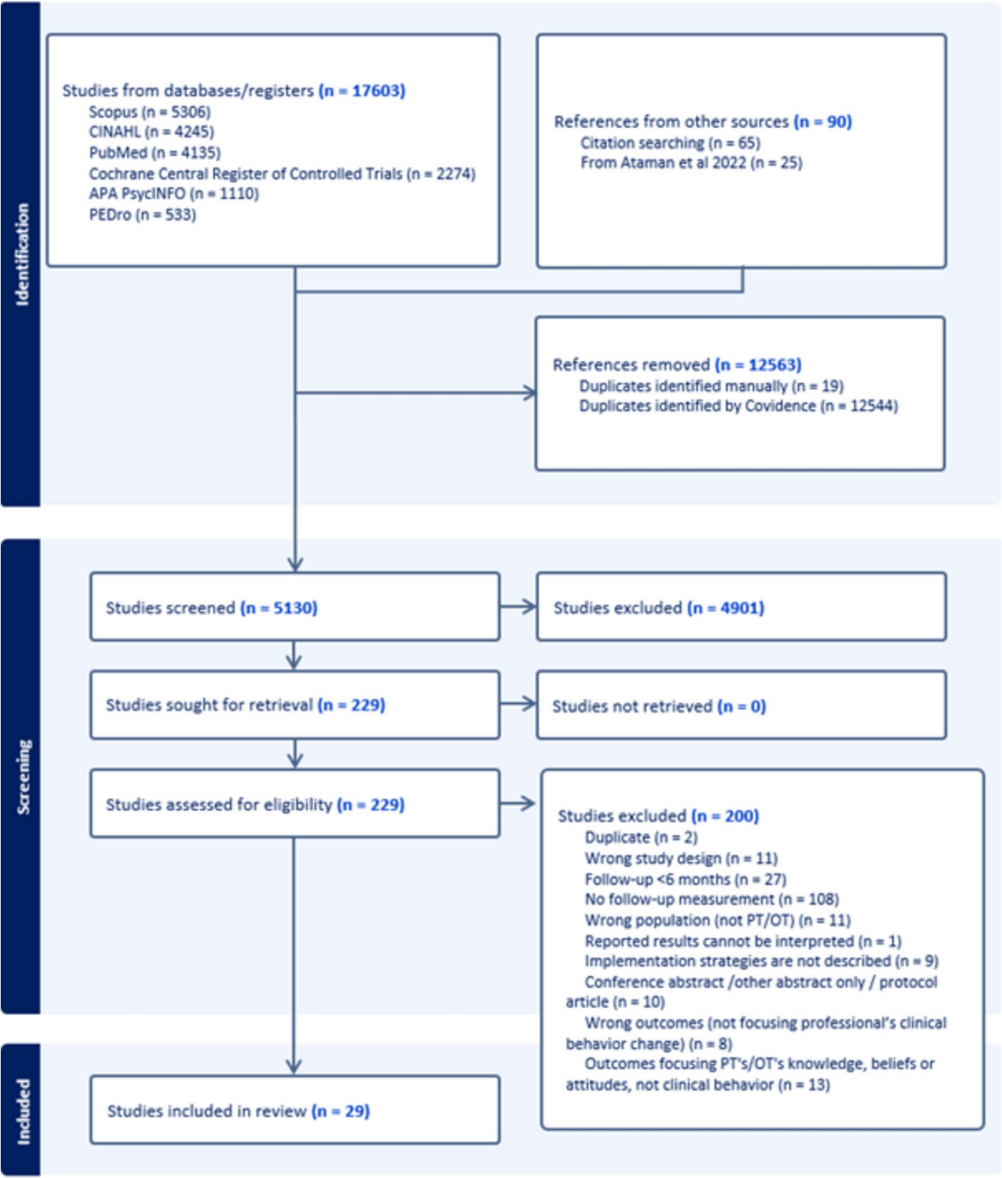
PRISMA chart for the study selection process

Figure 1 presents the PRISMA chart for the study selection process and number of studies. In the chart, the total number of items retrieved per database is reported. This means that the retrieved references per database from the initial search and the updated search are combined. In total, 29 studies were included in this scoping review.

All studies were conducted in Western countries (5 in Canada, 7 in the USA, 10 in Europe (Sweden, Norway, Netherlands, Switzerland, UK), 5 in Australia, and 2 in Israel). Most of the studies were conducted in primary health care settings (*n* = 10). The context of 8 studies was rehabilitation hospitals, and others were in pediatric settings (5), acute hospitals (3), elderly care (1), and outpatient orthopedic settings (1). One study was conducted in a continuing education setting where the participants came from different workplaces. Twenty-four of the studies used an experimental pre and post measurement design without a control group, and 5 used a control group. One study employed an RCT design. In addition to quantitative data, 3 studies collected qualitative data. Eighteen studies included exclusively PTs and 2 studies included exclusively OTs. One study included both PTs and OTs, and in 8 studies, other professionals, such as physicians and nurses, were included in addition to PTs and OTs.

Of the 29 studies measuring the clinical behaviors of professionals, 21 used self-reported outcome measures, such as questionnaires and surveys. In 10 studies, medical chart reviews were conducted. Only two studies utilized observation in clinical practice, and in one study, patient-reported data were collected. Twenty-one studies reported sustained changes in the clinical behavior of PTs and OTs, and eight studies reported unsustained changes. Among the studies reporting sustained changes in clinical behavior, sustainability was measured after 18 months up to several years in half of the studies compared to none among the studies reporting unsustained changes (Table 2).

**Table 2:** Follow-up time for studies reporting sustained and unsustained changes in clinical behavior

	6–8 m	12 m	18 m	24 m	48 m	3–6 y
Studies reporting sustained results	[35–41]	[42–45]	[46–48]	[49–53]	[54]	[55]
Studies reporting unsustained results	[56–59]	[12, 60–62]	-	-	-	-

m = months, y = years

### Implementation support strategies used in studies investigating the sustainability of PTs’ and OTs’ clinical behavior

Forty-three percent (9 out of 21) of the studies reporting sustained clinical behavior used 12–24 months of implementation support, compared to none of the studies reporting unsustained clinical behavior. Among the studies with unsustained changes, the implementation period was a maximum of 8 months (Table 3).

**Table 3:** The implementation support period for studies reporting sustained and unsustained changes in clinical behavior

	2 h-2 d	2–8 w	6–8 m	12–24 m	Unclear
Studies reporting sustained results	[36, 37, 41, 43, 44, 53]	[38]	[35, 39, 40, 48]	[42, 45–47, 49–52, 54]	[55]
Studies reporting unsustained results	[59, 60]	[58, 62]	[12, 56, 57]	-	[61]

h = hours, d = days, w = weeks, m = months

Twenty-two different implementation support strategies were identified in the studies (Table 4). Studies reporting sustained clinical behavior change used a median of 7 (range 3–12) implementation support strategies compared to 5 (range 2–10) in the studies reporting unsustained changes (see Additional file 5). The most commonly used implementation support strategies are presented in Table 4 and categorized according to the COM-B system [15] in Table 5.

**Table 4:** Implementation support strategies used in studies reporting sustained and unsustained changes in clinical behavior

Implementation support strategy	Studies reporting sustained results	Studies reporting unsustained results
Educational meetings (lectures, meetings, workshop)	[35–55]^a^	[12, 56–61]^a^
Audit and feedback	[35, 37–42, 44–46, 49–55]^a^	[12, 57]
Training/practice	[35, 36, 38, 39, 41–45, 48, 50–53]^a^	[12, 56, 57, 59, 60, 62]^a^
Resources (templates, patient information, access to databases, IT-support)	[35, 36, 38, 40, 42–44, 48, 50, 51, 53, 54]^a^	[12, 57, 61]
Problem solving	[35, 38, 40, 42–44, 47, 50, 51, 53, 55]^a^	[57]
Demonstration/modeling	[38, 41, 42, 44, 45, 47, 50–53, 55]^a^	[12, 56, 61]
Educational materials/Written information/Instructions	[35, 38–43, 49, 52, 53]	[12, 56, 58, 61]^a^
Communities of practice (case conferences, journal club, small group work, peer assessment/support)	[35, 38–40, 44, 46–49, 51, 54]^a^	[12, 57, 58]
Local opinion leaders/knowledge brokers/champions	[37, 38, 40, 42, 46, 48, 49, 51, 55]	[59, 61, 62]
Reminders	[38, 40, 42, 45, 50, 51]	-
Environmental modifications	[37, 44, 50–52, 54]	-
Mentoring/one-on-one support	[42, 47–49, 55]	[57, 58]
Goal setting	[35, 37, 51, 54]	[12, 57]
Organizational support (process changes)	[37, 47, 51, 54]	-
Leadership support	[39, 45, 54]	[12]
Action plan	[35, 47]	-
Local consensus processes	[37, 50]	-
Rewards	[54]	-
Support to local opinion leaders/knowledge brokers/champions	[55]	-
Financial resources	[48]	-
Monitoring the performance of the delivery of health care	[45]	[12]
Written assignments	-	[58]

^a^Implementation support strategies used in at least 50% of the studies reporting sustained or unsustained changes in clinical behavior

**Table 5:** Implementation support strategies used in at least half of the studies reporting sustained and unsustained changes in clinical behavior, categorized according to the COM-B system [15]

**Capability**	**Studies reporting sustained results**	**Studies reporting unsustained results**
Educational meetings (lectures, meetings, workshop)	X	X
Training/practice	X	X
Demonstration/modeling	X	
Audit and feedback		
Educational materials/Written information/Instructions		X
Communities of practice (case conferences, journal club, small group work, peer assessment/support)	X	
Mentoring/one-on-one support		
Local consensus processes		
Monitoring the performance of the delivery of health care		
**Motivation**		
Goal setting		
Action plan		
Rewards		
Reminders		
Written assignments		
**Opportunity**		
Resources (templates, patient information, access to databases, IT-support)	X	
Problem solving	X	
Local opinion leaders/knowledge brokers/champions		
Environmental modifications		
Organizational support (process changes)		
Leadership support		
Support to local opinion leaders/knowledge brokers/champions		
Financial resources		

Sustainability support strategies were explicitly mentioned to the same extent in both the studies reporting sustained results (7 out of 21 studies) and in the studies reporting unsustained results (3 out of 8 studies). In median two sustainability support strategies (range 1–5) were used per study. Audit and feedback and mentoring were the most commonly used strategies in the studies reporting sustained results (both used in three studies), and resources and action plans were the most commonly used (both used in two studies) in the studies reporting unsustained results.

The implementation strategies were theory-based in 86% (18 out of 21) of the studies reporting sustained results compared to 37.5% (3 out of 8) of the studies reporting unsustained results. In total, 17 theories were used in the included studies (Table 6). The knowledge-to-action framework was used in half of the studies reporting sustained results compared to none in the studies reporting unsustained results. Five of the studies reporting sustained results and two of the studies reporting unsustained results used more than one theory.

**Table 6:** Theories used in studies reporting sustained or unsustained changes in clinical behavior

Theory	Studies reporting sustained results	Studies reporting unsustained results
Knowledge to Action Framework	[37–40, 42, 48, 50, 51, 54, 55]	
Theoretical Domains Framework	[37, 42, 52, 54]	
Behavior Change Wheel	[42, 52]	
Social Cognitive Theory	[35, 39]	[12, 57]
COM-B	[52]	
RE-AIM QuEST	[43]	
The Implementation Model of Grol and Wensing	[46]	
CFIR	[50]	
Framework of Carroll & co	[36]	
Tropman’s organizational rubrik	[47]	
Control Theory	[44]	
Adult learning theories	[39]	[57]
Kirkpatrick’s taxonomy of training criteria	[41]	
PARIHS framework		[12]
Diffusion of innovations		[57]
Transtheoretical model		[57]
Experiential learning theory		[58]
No theory	[45, 49, 53]	[56, 59–62]

### Behavior change techniques used in studies investigating the sustainability of PTs’ and OTs’ clinical behavior

Two of the studies [42, 52] defined implementation support strategies in terms of behavior change techniques [34]. Twenty-three different behavior change techniques were identified in the studies (Table 7). The studies reporting sustained clinical behavior change used a median of 6 (range 3 to 11) behavior change techniques, and the studies reporting unsustained clinical behavior change used a median of 2.5 (range 2–10) behavior change techniques (see Additional file 6). The most commonly used behavior change techniques are presented in Table 7.

**Table 7:** Behavior change techniques (BCTs) used in studies reporting sustained or unsustained changes in clinical behavior. The BCTs are numbered according to Michie et al. [34]

BCT	Studies reporting sustained results	Studies reporting unsustained results
4.1 Instruction on how to perform the behavior	[35–48, 50–53]^a^	[12, 56–62]^a^
8.1 Behavioral practice/rehearsal	[35–39, 41–45, 47, 51–53]^a^	[12, 56–58, 60, 62]^a^
1.2 Problem solving	[35, 38, 40, 43, 44, 47–51, 53–55]^a^	[12, 57, 58]
6.1 Demonstration of the behavior	[35, 38, 41–44, 47, 50–53, 55]^a^	[12, 56, 57]
3.1 Social support (unspecified)	[38–40, 45–51]^a^	[12, 59, 61]
2.2 Feedback on behavior	[35, 41, 42, 46, 49, 50, 52–55]	[12, 57, 58]
7.1 Prompts/cues	[40, 42, 45, 49–52, 54]	[57]
12.1 Restructuring the physical environment	[37, 39, 42, 45, 50–52, 54]	-
12.5 Adding objects to the environment	[38, 40, 43, 44, 50, 52–54]	[12]
2.7 Feedback on outcome(s) of behavior	[37, 40, 45, 51, 54]	-
1.3 Goal setting (outcome)	[35, 37, 45, 51]	[12, 57]
2.3 Self-monitoring of behavior	[42, 48, 52]	[12]
1.4 Action planning	[35, 47]	-
3.2 Social support (practical)	[37, 55]	[57]
5.1 Information about health consequences	[42, 52]	-
1.6 Discrepancy between current behavior and goal	[35]	-
3.3 Social support (emotional)	[37]	-
4.2 Information about antecedents	[36]	-
9.1 Credible source	[52]	-
10.4 Social reward	[45]	-
15.1 Verbal persuasion about capability	[42]	-
15.3 Focus on past success	[52]	-
1.5 Action planning	-	[12]

^a^Behavior change techniques (BCTs) used in at least 50% of the studies reporting sustained or unsustained changes in clinical behavior

## Discussion

The aim of this scoping review was to explore the nature of studies investigating the sustainability of PTs’ and OTs’ clinical behavior when implementing evidence-based methods in health care. Our results show several differences between studies that have reported sustained and unsustained changes in the clinical behavior of PTs and OTs concerning the implementation period, the number of and most commonly used implementation support strategies and behavior change techniques, and the use of theory.

Similar to previous reviews [63, 64] about implementation interventions, based on our results, it seems that longer-term implementation support may be important not only for achieving behavioral changes among health care professionals but also for supporting the sustainability of these results. Separate sustainability strategies were seldom reported in the included studies, and there was no difference in the reporting of sustainability strategies between studies with sustained and unsustained results. Often, sustainability strategies are intertwined with the original implementation strategies and are therefore difficult to separate. Ataman et al. found that intermediate outcomes, for example, in the form of demonstrated outcomes, can act as a context for later outcomes [3].

A clear classification to determine the complexity of the interventions to be implemented is lacking [65] and therefore we were not able to classify the interventions to be implemented. However, our impression was that the interventions to be implemented varied between fairly simple (e.g., hand hygiene compliance and the use of outcome measures) and complex (e.g., stratified primary care models for low back pain and behavioral medicine approaches in physiotherapy) methods. It seems like many studies that have achieved sustained results aimed at implementing a fairly simple object, such as a standardized test or a measurement instrument. A classification to determine the complexity of the interventions to be implemented would be helpful for understanding effective implementation and sustainability strategies, as there may be differences between the best strategies for implementing objects of differing complexity. It also needs to be remembered that implementation support is often resource intensive, and whether the efforts truly pay off must be evaluated.

It is known that there is an obvious difference between observed and self-reported clinical behavior, and professionals often overestimate their use of target behaviors [25]. It is also considered that observation is a valid method for assessing clinical practice [66]. Many studies did not seem to use clinical behaviors as outcome measures, and we excluded 13 studies because they only measured the knowledge and attitudes of professionals. The included studies reported measuring clinical behaviors, but most of those studies were based on questionnaires and chart reviews. Only two studies used observation of practice as a measure of clinical behavior. Our results again highlight the importance of reviewing how we evaluate clinical practice in implementation studies.

It is recommended to use a combination of theories in implementation research to obtain a more complete understanding of the implementation process [14]. However, most of the interventions implemented in the studies included in our review were based on only one theory. Seventeen different theories were referred to in the included studies, and the knowledge-to-action framework [67] was the most commonly used theory. It was obvious that a theory-based implementation support strategy based on the knowledge-to-action framework was a characteristic of the interventions reporting sustained results. Davis et al. [68] noted that the way in which the implementation was guided by theory was difficult to determine because the description of the application of theory was usually rather vague. We agree on that, regarding the studies included in this scoping review. Thus, the impact mechanisms and effects of using the knowledge-to-action framework in implementation need to be further evaluated. Nilsen [14] describes the knowledge-to-action framework as a process model that is used to guide the implementation process by offering practical guidance in planning and executing the implementation. He also describes other theoretical approaches aimed at understanding or explaining what influences implementation outcomes (determinant frameworks) and evaluating implementation (evaluation frameworks). However, these theoretical approaches were seldom used in the included studies.

The sustainability of implementation is dependent on professionals’ behavioral change and their ability to maintain this behavior change. When reviewing the most commonly used implementation strategies in our included studies, i.e., the implementation support strategies used in at least 50% of the studies, most of them targeted capability. None targeted motivation, and there was no difference between the studies reporting sustained results and the studies reporting unsustained results regarding the extent to which these conditions were used. However, implementation strategies targeting opportunity differ between the groups. The most common implementation strategies in studies reporting sustained results targeted opportunity, but not in studies reporting unsustained results. An explanation for why some implementation efforts achieves sustained results and others do not may be how well the intervention covers all the conditions in the COM-B system.

Our results indicate that the use of multiple implementation support strategies and multiple behavior change techniques may help health care professionals sustain their intended behaviors. A previous review recommended the use of multiple behavior change strategies [69], but others have not found differences between single and multifaceted interventions in changing clinicians’ behavior in the short term [70]. Some suggestions have been made about what strategies should be used (e.g., local opinion leaders, monitoring performance, peer assessment, case studies, practical tools) [1, 63] in the initial behavior change phase. A review by Grimhaw et al. showed that the effectiveness of individual implementation support strategies usually varies between 4.2% and 6.0%. However, the “local opinion leader” strategy was outstanding, showing an effectiveness of 12.0% [71]. In the initial behavior change phase, no single intervention seems to be superior, and we have much less knowledge about what is important in sustaining the new behavior. We did not find any patterns of behavior change techniques separating studies that received sustained and unsustained results. The reporting of the strategies used varied a lot. As only two studies used the classification of behavior change techniques [34], there is room for improvement to make the implementation and sustainability strategies easier to follow.

Implementation studies are often conducted separately from randomized controlled trials measuring patient outcomes. It would be interesting to have more studies combining both to be able to estimate the effect of the intervention and whether the level of training and implementation support of clinicians delivering the intervention has an impact on patient outcomes. Often, in RCTs, clinician training is poorly reported, and competency and fidelity testing are not performed [72]. Therefore, we do not actually know whether the intended research intervention was performed as planned. Despite having the necessary tools, clinicians often fail to deliver the optimum evidence-based treatment. The phenomenon of therapist drift, our tendency to drift away from evidence-based practice, may be an important reason why therapies are often less effective than they should be in the clinical practice. The reasons for therapist drift are complex; therefore, multifaceted approaches are needed to change our practice [73].

### Strengths and limitations

It was challenging to identify studies reporting on the outcome sustainability of this review. Most studies do not use the concept “sustainability” or similar in the title or abstract. This is something that other reviews on sustainable implementation have also found [7]. To include potentially relevant studies, the search string concerning sustainability therefore contained numerous words that can be related in different ways to sustainable interventions. Additionally, search terms concerning the review’s intervention and implementation of evidence-based methods entailed a broad search strategy, as the concept can be described with different terms. McKibbon et al. [74] showed how elusive the subject is when they found 100 words that somehow relate to knowledge translation in their work to develop a search filter. Thus, the search strategy itself could not identify studies reporting on sustainability, which meant that we had to screen a large number of studies, and most of the studies reporting clinical behavior change needed to be reviewed in full text. By excluding qualitative studies, the validation of assessment of clinical practice were supposed to increase. It is also possible that we, by excluding these studies, missed an opportunity to capture relevant explanations about sustained clinical behavior. Since this was not the scope of this study, we welcome future studies in this area. It is possible that we missed some studies that should have been included in this scoping review. However, the strategy to include if we were unsure during the review process and the strategy to use two reviewers working separately strengthened the review process.

The interpretation of the term sustainability is problematic. How compliant does the professional need to be to the intervention to be implemented to consider the clinical behavior as sustained? When evaluating if there is a clinical behavior change, statistical analysis is often used. Based on the p-value, the clinical behavior is interpreted as changed or not. If the change still remains after at least six months, it is considered as sustained. However, this analysis only shows if there is a change in clinical behavior, but not if that change is enough to have any effect on the patient. Therefore, evaluations of sustainability of clinical behavior change also needs to include patient outcomes.

In this scoping review, the implementation support strategies used in the included studies are, when possible, named in accordance with the EPOC group taxonomy [10]. However, half of the implementation support strategies did not fit into the taxonomy and were named differently. This shows a limitation in the EPOC taxonomy, but it also means that not all implementation support strategies are described based on a standardized model. It is also possible that there is some overlap between the strategies; for example, educational meetings may overlap with problem solving and demonstrations. To ensure transparency, the implementation support strategies are described as concretely as possible and presented in Additional file 5.

When translating the implementation support strategies to behavior change techniques, we interpreted the description of the implementation support strategies in relation to the behavior change technique taxonomy [34]. Only two of the studies defined the implementation support strategies in terms of behavior change techniques. Even though they had defined the behavior change techniques used, these techniques did not fully match the description of the implementation support strategies. According to our interpretation, more techniques were used than what was stated in the study, which we also presented in Additional file 6. Implementation support strategies are usually poorly reported in implementation studies as well as in controlled trials [72]. Some strategies were therefore more challenging to interpret. Local opinion leader was interpreted as 3.1 social support (unspecified) since it was unclear what kind of social support they were given. Communities of practice were interpreted as one or several out of four behavior change techniques since they could concern 1.2 problem solving, 2.2 feedback, and different kinds of social support (3.1 unspecified, 3.2 practical). However, in some studies, the communities of practice were only described as “small group work”, which could mean many different things and should perhaps be interpreted as something else. Educational meetings were usually not described and were mostly interpreted as 4.1 instructions on how to perform a behavior but also as 4.2 information about antecedents and 5.1 information about health consequences. It is possible that we missed some behavior change technique or included some that should not be present. To ensure transparency, the translation of implementation support strategies to behavior change techniques is presented in Additional file 5.

## Conclusions

Studies reporting sustained results of PTs’ and/or OTs’ clinical behavior used longer implementation periods, more implementation strategies, more theory-based interventions, and more behavior change techniques. Audit and feedback, resources, problem solving, demonstrations, and communities of practice were the most frequently used implementation strategies in studies with sustained results. The corresponding behavior change techniques were problem solving, demonstration of behavior, and social support. Our study also highlights the importance of well-described implementation studies, including accurate descriptions of both the clinical intervention that will be implemented and the implementation support strategies. Describing implementation support strategies as standardized behavior change techniques is one way to enrich the description. To evaluate the sustainability of implementation efforts, professionals’ clinical behavior must be investigated with validated methods.

## Supplementary Information

Supplementary Material 1.

Supplementary Material 2.

Supplementary Material 3.

Supplementary Material 4.

Supplementary Material 5.

Supplementary Material 6.

## Data Availability

All data generated or analyzed during this study are included in this published article and its supplementary information files.

## Abbreviations

OT: Occupational therapist
PT: Physiotherapist
