# Supplementary material for: Sustainable implementation efforts in physio- and occupational therapy: a scoping review
Source: Implement Sci Commun. 2024 Dec 12;5:138. doi: 10.1186/s43058-024-00676-8 (PMC11636039; doi:10.1186/s43058-024-00676-8)
Supplement: Supplementary file 2 — Supplementary Material 2. [file 43058_2024_676_MOESM2_ESM.docx]

Additional file 2. Search strategy

### **PubMed (pubmed.ncbi.nlm.nih.gov)**

| **Search #** | *Search terms* |
| --- | --- |
|  | "implement*"[Title/Abstract] OR uptake[Title/Abstract] OR diffusion[Title/Abstract] OR disseminat*[Title/Abstract] OR adopt*[Title/Abstract] OR "knowledge transl*"[Title/Abstract] OR "continuing education"[Title/Abstract] OR "evidence-informed"[Title/Abstract] OR "evidence-based"[Title/Abstract] OR "evidence based"[Title/Abstract] OR "Evidence-Based Practice"[MeSH Terms] OR "Implementation Science"[MeSH Terms] |
|  | "physical therapist*"[Title/Abstract] OR "physiotherapist*"[Title/Abstract] OR "occupational therapist*"[Title/Abstract] OR "Occupational Therapists"[MeSH Terms] OR "Physical Therapists"[MeSH Terms] |
|  | "implementation outcome*"[Title/Abstract] OR "institutionalisation"[Title/Abstract] OR "institutionalization"[Title/Abstract] OR "normalization"[Title/Abstract] OR "normalisation"[Title/Abstract] OR "re-invention"[Title/Abstract] OR "continued use"[Title/Abstract] OR "assimilation"[Title/Abstract] OR "long term use"[Title/Abstract] OR "use long term"[Title/Abstract] OR "program continuation"[Title/Abstract] OR "implementation continuation"[Title/Abstract] OR "follow-up"[Title/Abstract] OR "evaluat*"[Title/Abstract] OR "before-after"[Title/Abstract] OR "before-and-after"[Title/Abstract] OR "maintain*"[Title/Abstract] OR "sustain*"[Title/Abstract] OR "durability"[Title/Abstract] OR "routinization"[Title/Abstract] OR "routinisation"[Title/Abstract] OR "continuation"[Title/Abstract] OR "Policy Compliance"[Title/Abstract] OR "Protocol Compliance"[Title/Abstract] OR "Institutional Adherence"[Title/Abstract] OR "Guideline Adherence"[Title/Abstract] OR "Guideline Adherence"[MeSH Terms] |
|  | #1 AND #2 AND #3 |
| Limits | Filters applied: English. |
| **Totalt** | Result numbers and search dates: 1,984 results on January 12, 2023; 2,151 results on October 12, 2023) |

### **Cinahl Plus (EBSCOhost)**

| **Search #** | *Search terms* |
| --- | --- |
|  | (TI implement* OR AB implement*) OR (TI uptake OR AB uptake) OR (TI diffusion OR AB diffusion) OR (TI disseminat* OR AB disseminat*) OR (TI adopt* OR AB adopt*) OR (TI "knowledge transl*" OR AB "knowledge transl*") OR (TI "continuing education" OR AB "continuing education") OR (TI evidence-informed OR AB evidence-informed) OR (TI evidence-based OR AB evidence-based) OR (TI "evidence based" OR AB "evidence based") OR (MH "Professional Practice, Evidence-Based+") OR (MH "Implementation Science") |
|  | (TI "physical therapist*" OR AB "physical therapist*") OR (TI physiotherapist* OR AB physiotherapist*) OR (TI "occupational therapist*" OR AB "occupational therapist*") OR (MH "Occupational Therapists") OR (MH "Physical Therapists") |
|  | (TI "implementation outcome*" OR AB "implementation outcome*") OR (TI institutionalisation OR AB institutionalisation) OR (TI institutionalization OR AB institutionalization) OR (TI normalization OR AB normalization) OR (TI normalisation OR AB normalisation) OR (TI re-invention OR AB re-invention) OR (TI "continued use" OR AB "continued use") OR (TI assimilation OR AB assimilation) OR (TI "long term use" OR AB "long term use") OR (TI "use long term" OR AB "use long term") OR (TI "program continuation" OR AB "program continuation") OR (TI "implementation continuation" OR AB "implementation continuation") OR (TI follow-up OR AB follow-up) OR (TI evaluat* OR AB evaluat*) OR (TI before-after OR AB before-after) OR (TI before-and-after OR AB before-and-after) OR (TI maintain* OR AB maintain*) OR (TI sustain* OR AB sustain*) OR (TI durability OR AB durability) OR (TI routinization OR AB routinization) OR (TI routinisation OR AB routinisation) OR (TI continuation OR AB continuation) OR (TI "Policy Compliance" OR AB "Policy Compliance") OR (TI "Protocol Compliance" OR AB "Protocol Compliance") OR (TI "Institutional Adherence" OR AB "Institutional Adherence") OR (TI "Guideline Adherence" OR AB "Guideline Adherence") OR (MH "Guideline Adherence") |
|  | #1 AND #2 AND #3 |
| Limits | Narrow by Language: - english |
| **Total** | Result numbers and search dates: 2,075 results on January 12, 2023; 2,170 results on October 12, 2023) |

### **Scopus (www.scopus.com)**

| **Search #** | *Search terms* |
| --- | --- |
|  | TITLE-ABS ( implement* ) OR TITLE-ABS ( uptake ) OR TITLE-ABS ( diffusion ) OR TITLE-ABS ( disseminat* ) OR TITLE-ABS ( adopt* ) OR TITLE-ABS ( "knowledge transl*" ) OR TITLE-ABS ( "continuing education" ) OR TITLE-ABS ( evidence-informed ) OR TITLE-ABS ( evidence-based ) OR TITLE-ABS ( "evidence based" ) OR INDEXTERMS ( "Evidence-Based Practice" ) OR INDEXTERMS ( "Implementation Science" ) |
|  | TITLE-ABS ( "physical therapist*" ) OR TITLE-ABS ( physiotherapist* ) OR TITLE-ABS ( "occupational therapist*" ) OR INDEXTERMS ( "Occupational Therapists" ) OR INDEXTERMS ( "Physical Therapists" ) |
|  | TITLE-ABS("implementation outcome*") OR TITLE-ABS(institutionalisation) OR TITLE-ABS(institutionalization) OR TITLE-ABS(normalization) OR TITLE-ABS(normalisation) OR TITLE-ABS(re-invention) OR TITLE-ABS("continued use") OR TITLE-ABS(assimilation) OR TITLE-ABS("long term use") OR TITLE-ABS("use long term") OR TITLE-ABS("program continuation") OR TITLE-ABS("implementation continuation") OR TITLE-ABS(follow-up) OR TITLE-ABS(evaluat*) OR TITLE-ABS(before-after) OR TITLE-ABS(before-and-after) OR TITLE-ABS(maintain*) OR TITLE-ABS(sustain*) OR TITLE-ABS(durability) OR TITLE-ABS(routinization) OR TITLE-ABS(routinisation) OR TITLE-ABS(continuation) OR TITLE-ABS("Policy Compliance") OR TITLE-ABS("Protocol Compliance") OR TITLE-ABS("Institutional Adherence") OR TITLE-ABS("Guideline Adherence") OR INDEXTERMS("Guideline Adherence") |
|  | #1 AND #2 AND #3 |
| Limits | ( LIMIT-TO ( LANGUAGE , "English" ) ) |
| **Total** | Result numbers and search dates:  2,547 results on January 12, 2023; 2,759 results on October 12, 2023) |

###

### **Cochrane Central Register of Controlled Trials (www.cochranelibrary.com/central)**

| **Search #** | *Search terms* |
| --- | --- |
|  | implement*:ti,ab OR uptake:ti,ab OR diffusion:ti,ab OR disseminat*:ti,ab OR adopt*:ti,ab OR ("knowledge" NEXT transl*):ti,ab OR "continuing education":ti,ab OR evidence-informed:ti,ab OR evidence-based:ti,ab OR "evidence based":ti,ab OR [mh "Evidence-Based Practice"] OR [mh "Implementation Science"] |
|  | ("physical" NEXT therapist*):ti,ab OR physiotherapist*:ti,ab OR ("occupational" NEXT therapist*):ti,ab OR [mh "Occupational Therapists"] OR [mh "Physical Therapists"] |
|  | ("implementation" NEXT outcome*):ti,ab OR institutionalisation:ti,ab OR institutionalization:ti,ab OR normalization:ti,ab OR normalisation:ti,ab OR re-invention:ti,ab OR "continued use":ti,ab OR assimilation:ti,ab OR "long term use":ti,ab OR "use long term":ti,ab OR "program continuation":ti,ab OR "implementation continuation":ti,ab OR follow-up:ti,ab OR evaluat*:ti,ab OR before-after:ti,ab OR before-and-after:ti,ab OR maintain*:ti,ab OR sustain*:ti,ab OR durability:ti,ab OR routinization:ti,ab OR routinisation:ti,ab OR continuation:ti,ab OR "Policy Compliance":ti,ab OR "Protocol Compliance":ti,ab OR "Institutional Adherence":ti,ab OR "Guideline Adherence":ti,ab OR [mh "Guideline Adherence"] |
|  | #1 AND #2 AND #3 |
| Limits | - |
| **Total** | Result numbers and search dates:  1,086 results on January 12, 2023; 1,188 results on October 12, 2023) |

### **APA PsycInfo (EBSCOhost)**

| **Search #** | *Search terms* |
| --- | --- |
|  | DE "Evidence Based Practice" OR ( (TI implement* OR AB implement*) OR (TI uptake OR AB uptake) OR (TI diffusion OR AB diffusion) OR (TI disseminat* OR AB disseminat*) OR (TI adopt* OR AB adopt*) OR (TI "knowledge transl*" OR AB "knowledge transl*") OR (TI "continuing education" OR AB "continuing education") OR (TI evidence-informed OR AB evidence-informed) OR (TI evidence-based OR AB evidence-based) OR (TI "evidence based" OR AB "evidence based") ) |
|  | ( (DE "Physical Therapists") OR (DE "Occupational Therapists") ) OR ( (TI "physical therapist*" OR AB "physical therapist*") OR (TI physiotherapist* OR AB physiotherapist*) OR (TI "occupational therapist*" OR AB "occupational therapist*") ) |
|  | (TI "implementation outcome*" OR AB "implementation outcome*") OR (TI institutionalisation OR AB institutionalisation) OR (TI institutionalization OR AB institutionalization) OR (TI normalization OR AB normalization) OR (TI normalisation OR AB normalisation) OR (TI re-invention OR AB re-invention) OR (TI "continued use" OR AB "continued use") OR (TI assimilation OR AB assimilation) OR (TI "long term use" OR AB "long term use") OR (TI "use long term" OR AB "use long term") OR (TI "program continuation" OR AB "program continuation") OR (TI "implementation continuation" OR AB "implementation continuation") OR (TI follow-up OR AB follow-up) OR (TI evaluat* OR AB evaluat*) OR (TI before-after OR AB before-after) OR (TI before-and-after OR AB before-and-after) OR (TI maintain* OR AB maintain*) OR (TI sustain* OR AB sustain*) OR (TI durability OR AB durability) OR (TI routinization OR AB routinization) OR (TI routinisation OR AB routinisation) OR (TI continuation OR AB continuation) OR (TI "Policy Compliance" OR AB "Policy Compliance") OR (TI "Protocol Compliance" OR AB "Protocol Compliance") OR (TI "Institutional Adherence" OR AB "Institutional Adherence") OR (TI "Guideline Adherence" OR AB "Guideline Adherence") |
|  | #1 AND #2 AND #3 |
| Limits | Narrow by Language: - english |
| **Total** | Result numbers and search dates:  533 results on January 12, 2023; 577 results on October 12, 2023) |

### **PEDro (pedro.org.au)**

| **Search #** | *Search terms* |
| --- | --- |
|  | implement* AND guideline* |
| Limits | - |
| **Total** | Result numbers and search dates:  262 results on January 12, 2023; 271 results on October 12, 2023) |
