# Supplementary material for: Sustainable implementation efforts in physio- and occupational therapy: a scoping review
Source: Implement Sci Commun. 2024 Dec 12;5:138. doi: 10.1186/s43058-024-00676-8 (PMC11636039; doi:10.1186/s43058-024-00676-8)
Supplement: Supplementary file 3 — Supplementary Material 3. [file 43058_2024_676_MOESM3_ESM.docx]

Additional file 3. Data extraction – study characteristics

| **Author, year** | **Title** | **Aim** | **Country** | **Design** | **Setting** | **Participants, N pre/follow-up** | **Intervention to be implemented** |
| --- | --- | --- | --- | --- | --- | --- | --- |
| Auld M, Johnston L. 2019 | Getting inTOUCH: Outcomes of a knowledge translation intervention for tactile assessment knowledge, barriers, and practice in paediatric therapists working with children with cerebral palsy | This study aims to use the KTA framework to guide the development of a multi-faceted intervention for improving the knowledge, skills, and implementation of tactile assessments by paediatric therapists. By linking the known barriers to completing tactile assessments to successful behaviour change techniques we aim to effectively improve the uptake of tactile assessment amongst a group of paediatric therapists. | Sweden | Experimental pre-post, no control group | Pediatric | PT, OT  Pre: 12 12m: 8 | Practice of tactile assessment: Touch-in-10 and Touch-in-2. |
| Barton C, Kemp J, Roos E, Skou S, Dundules K, Pazzinatto M, Francis M, Lanning N, Wallis J, Crossley K. 2021 | Program evaluation of GLA:D® Australia: Physiotherapist training outcomes and effectiveness of implementation for people with knee osteoarthritis | In this paper, we evaluate the implementation of GLA:D® within an Australian context. Guided by the RE-AIM QuEST (Reach Effectiveness Adoption Implementation Maintenance Qualitative Evaluation for Systematic Translation) framework, this program evaluation investigated (i) changes in physiotherapists' practices, and confidence and beliefs about capabilities to provide patient education and exercise-therapy to people with knee osteoarthritis; (ii) outcomes of people with knee osteoarthritis participating in the program, including primary (pain, knee- and health-related quality of life), and secondary(surgical desire, functional performance) measures; and (iii) barriers and enablers to reach, implementation, adoption and maintenance of the program in Australian public and private settings | Australia | Experimental pre-post, no control group | Primary health care | PT  Pre: 1064 12m: 293 | The Good Life with osteoArthritis from Denmark (GLA:D®) program. |
| Carlfjord S, Landén Ludvigsson M, Peolsson A, Peterson G.  2021 | Adoption of a research-based program for neck disorders implemented in primary care physiotherapy: a short- and long-term follow-up survey study | The aim of the present study was to evaluate the adoption of a research-based diagnosis and exercise program for treatment of neck disorders, implemented among physiotherapists in primary care, in terms of self-reported behavior and confidence, three and 12 months after the education. | Sweden | Experimental pre-post, no control group | Primary health care | PT  Pre: 261 12m: 123 | Neck-specific exercise program based on research findings. |
| Fritz J, Wallin L, Söderlund A, Almqvist L, Sandborgh M  2020 | Implementation of a behavioral medicine approach in physiotherapy: Impact and sustainability | The aim of this study was to explore the effects on and sustainability of physiotherapists’ clinical behavior when using facilitation to support the implementation of a behavioral medicine approach in primary health care for patients with persistent musculoskeletal pain. | Sweden | Experimental pre-post with control group | Primary health care | PT  IG Pre: 15, 6m: 12, 12m: 8  CG pre: 9 6m: 7, 12m: 6 | An evidence based behavioral medicine approach in physiotherapy. |
| Gross D, Lowe A  2009 | Evaluation of a knowledge translation initiative for physical therapists treating patients with work disability | We evaluated a knowledge translation and exchange (KTE) initiative aimed at providing physical therapists with best practice information regarding work disability prevention | Canada | Experimental pre-post, no control group | Primary health care | PT  Pre: 241 12m: 164 | Best practice resource guide for work disability prevention. |
| Kafri M, Levron Y, Atun-Einy O.  2023 | Assessing the impact of a knowledge translation intervention on physical therapists' self-efficacy and implementation of motor learning practice | a. To evaluate the influence of a “KT-ML” intervention  on ML-related self-efficacy, reported ML  implementation, and general perceptions and work  environment among certified PTs. b. To evaluate the long-term influence of the  intervention on ML-related self-efficacy, reported  ML implementation, and work environment in a  subsample of certified PTs. c. To evaluate the process of change and assimilation  immediately after the intervention and over time as  perceived by the PTs. d. To estimate the intervention from the participants’  perspective. | Israel | Experimental pre-post, no control group | Rehabilitation hospital | PT  Pre: 111  24 m: 25 | Systematic application of motor learning knowledge in clinical practice. |
| Karas S, Westerheide A, Daniel L.  2016 | A Knowledge Translation Programme to Increase the Utilization of Thoracic Spine Mobilization and Manipulation for Patients with Neck Pain | The purpose of the present research was to evaluate the effects of a structured knowledge translation programme on the frequency of manual therapy techniques performed by physical therapists on patients with neck pain | USA | Experimental pre-post, no control group | Primary health care | PT  Pre: 13  6m: 12 | Thoracic spine techniques for their patients with neck pain. |
| Lineker S, Bell M, Badley E.  2011 | Evaluation of an inter-professional educational intervention to improve the use of arthritis best practices in primary care | This report describes the evaluation of the national program and the lessons learned in implementation | Canada | Experimental pre-post, no control group | Primary health care | PT, OT, Nurses; Physicians; Others  Pre: 553 6m: 275 | The Getting a Grip on Arthritis© program, Clinical practice guidelines of rheumatoid arthritis and osteoarthritis. |
| McCluskey A, Lovarini M.  2005 | Providing education on evidence-based practice improved knowledge but did not change behaviour: a before and after study | The aim of this before and after study was to measure the effect of a multifaceted intervention on evidence-based practice, on the knowledge and skills, behaviour and attitudes of occupational therapists. | Australia | Experimental pre-post, no control group |  | OT  Pre: 114 8m: 51 | Evidence-based practice skills and knowledge. |
| McDonnell B, Stillwell S, Hart S, Davis R.  2018 | Breaking Down Barriers to the Utilization of Standardized Tests and Outcome Measures in Acute Care Physical Therapist Practice: An Observational Longitudinal Study | The purpose of this quality improvement (QI) effort was to implement a series of interventions aimed at increasing both use and interpretation of STOM by physical therapists practicing in acute care | Israel | Experimental pre-post, no control group | Acute hospital | PT  24-34 but nor specified how many at pre and 24m. | Standardized tests and outcome measures (STOM). |
| Meerhoff F, van Dulmen S, Maas M, Heijblom K, Nijhuis-van der Sanden M, Van der Wees P.  2017 | Development and Evaluation of an Implementation Strategy for Collecting Data in a National Registry and the Use of Patient-Reported Outcome Measures in Physical Therapist Practices: Quality Improvement Study | The aims of this article are to describe the process of the development and adaptation of the implementation strategy of the innovative quality policy and to evaluate the feasibility of 2 key elements—the implementation of PROMs measurements in physical therapist practices and the delivery of data by physical therapist practices to the registry. | Netherlands | Experimental pre-post, no control group | Primary health care | PT  Pre: 272 18m: 272 | Patient-reported outcome measures (PROMs) in physical therapist practices. |
| Moore J, Carpenter J, Doyle A, Doyle L, Hansen P, Hahn B, Hornby TJ, Roth H, Spoeri S, Tappan R, Van Der Laan K.  2018 | Development, Implementation, and Use of a Process to Promote Knowledge Translation in Rehabilitation | To examine the use and effect of the Battery of Rehabilitation Assessments and Interventions on evidence-based practice (EBP) over 6 years | Canada | Experimental pre-post, no control group | Rehabilitation hospital | PT, OT, Others  Pre: 136 3y: 115 6y: 121 | The Battery of Rehabilitation Assessments and Interventions on evidence-based practice (EBP). |
| Moore J, Bø E, Erichsen A, Rosseland I, Halvorsen J, Bratlie H, Hornby G, Nordvik JE.  2021 | Development and Results of an Implementation Plan for High-Intensity Gait Training | Using mix-methods research, including surveys and informal discussions, we evaluated current practice, barriers, outcomes, and the sustainability of high-intensity gait training in practice | Norway | Mixed methods | Rehabilitation hospital | PT  Pre: 9  24m: 10 | High-intensity gait training in stroke rehabilitation |
| Moore J, Virva R, Henderson C, Lenca L, Butzer J, Lovell L, Roth E, Graham I, Hornby G.  2022 | Applying the Knowledge-to-Action Framework to Implement Gait and Balance Assessments in Inpatient Stroke Rehabilitation | The overall objectives of this project were to implement and sustain use of a gait assessment battery (GAB) that included the Berg Balance Scale, 10-meter walk test, and 6-minute walk test during inpatient stroke rehabilitation. The study objective was to assess the effect of the study intervention on clinician adherence to the recommendations and its effect on clinician perceptions and the organization. | Canada | Experimental pre-post, no control group | Rehabilitation hospital | PT, Others  Pre: 8 48m: unclear | A gait assessment battery. |
| Moseng T, Dagfinrud H, Østerås N.  2019 | Implementing international osteoarthritis guidelines in primary care: uptake and fidelity among health professionals and patients | Was the uptake of patient information, exercise and referral to services for support on weight reduction higher among the intervention group compared to the control group? How was the fidelity to the implementation strategy and intervention in the study? | Norway | RCT | Primary health care | PT, Physicians  Pre: 77  6m: unclear | The SAMBA model for integrated osteoarthritis care. The model comprised a structured pathway for the patient through the health care system. |
| Novak I, McIntyre S.  2010 | The effect of Education with workplace supports on practitioners' evidence-based practice knowledge and implementation behaviours | The aim of this study was to test the hypothesis that the dual use of education and change management interventions (designed to remove EBP barriers and enhance EBP attitudes and implementation culture) would improve clinicians’ performance in answering EBP clinical questions and indirectly increase EBP utilisation behaviour. | Australia | Experimental pre-post, no control group | Pediatric | PT, OT, Physicians; Others  Pre: 88 18m: unclear | Evidence-based practice. |
| Olsen N, Bradley P, Espehaug B, Wammen Nortvedt M, Lygren H, Frisk B, Bjordal JM.  2015 | Impact of a multifaceted and clinically integrated training program in evidence-based practice on knowledge, skills, beliefs and behaviour among clinical instructors in physiotherapy: A non-randomized controlled study | The aim of this study was to assess the short- and long-term impact of a six-month multifaceted and clinically integrated training program in EBP on the knowledge, skills, beliefs and behaviour of CIs supervising physiotherapy students. | Norway | Experimental pre-post with control group | Elderly care | PT  IG Pre: 14, 6m: 13  CG Pre: 15, 6m: 9 | EBP knowledge, skills, beliefs and behavior. |
| Pöder U, Fogelberg-Dahm M, Wadensten B.  2011 | Implementation of a multi-professional standardized care plan in electronic health records for the care of stroke patients | The aim of the present study was to compare staff opinions about standardized care plans and self-reported habits with regard to documentation, and their perceived knowledge about the evidence-based guidelines for care and treatment (Socialstyrelsen 2005) and nursing (SSF 2005), before and after implementation of an evidence-based standardized care plan and quality standard for stroke care. The aim was also to describe staff opinions about, and their use of, the implemented evidence-based standardized care plan and quality standard. | Sweden | Experimental pre-post, no control group | Acute hospital | PT, OT, Nurses  Pre: 34, 12m: 37 | A standardized care plan, including the evidence-based quality standard. The system is an integrated electronic patient record intended for use by all authorized health professionals at a stroke unit. |
| Romney W, Salbach N, Parrott JS, Deutsch J.  2020 | A Knowledge Translation Intervention Designed and Implemented by a Knowledge Broker Improved Documented Use of Gait Speed: A Mixed-Methods Study | The purpose of this study was to determine whether an intervention tailored by an external KB, cocreated with the PTs and supported by the supervisor, would increase the use of gait speed by PTs working at an inpatient subacute rehabilitation hospital | USA | Experimental pre-post with control group; Mixed methods | Rehabilitation hospital | PT  Pre: 9-11  6 to 8m: 9-11 | To implement one outcome measure: 4MWT for patients older than 60 years. |
| Romney W, Wormley M, Veneri D, Oberlander A, Grevelding P, Rice J, Moore J.  2022 | Knowledge translation intervention increased the use of outcome measures by physical therapists in inpatient rehabilitation | The purpose of this study was to describe the outcome of a knowledge translation (KT) intervention to increase the use of outcome measures by physical therapists in an inpatient rehabilitation setting. | USA | Experimental pre-post, no control group | Rehabilitation hospital | PT  Pre: 14  24m: 14 | Improving the use and documented use of outcome measures within the facility: 10-Meter Walk Test, Berg Balance Scale, Timed Up and Go, Functional Reach Test (FRT) and Five Times Sit to Stand Test (FTSTS). Performing at least one of these outcome measure for every patient in the hospital at initial examination and discharge. |
| Russell D, Rivard L, Walter S, Rosenbaum P, Roxborough L, Cameron D, Darrah J, Bartlett D, Hanna S, Avery L.  2010 | Using knowledge brokers to facilitate the uptake of pediatric measurement tools into clinical practice: a before-after intervention study | The purpose of this study was to evaluate the impact of a multifaceted knowledge translation intervention, using PTs as knowledge brokers (KBs) to facilitate the use in clinical practice of four evidence-based measurement tools designed to evaluate and understand motor function in children with cerebral palsy (CP). | Canada | Experimental pre-post, no control group; Mixed methods | Pediatric | PT  Pre: 122  6.: 114  12m: 105  18m: 95 | Four evidence-based measurement tools designed to evaluate and understand motor function in children with cerebral palsy (CP): GMFCS, GMFM-88, GMFM-66, MGC. |
| Sakzewski L, Ziviani J, Boyd R  2016 | Translating Evidence to Increase Quality and Dose of Upper Limb Therapy for Children with Unilateral Cerebral Palsy: A Pilot Study | To pilot efficacy of a tailored multifaceted implementation program to change clinical practice of occupational therapists (OTs) providing up-per limb (UL) therapy for children with unilateral cerebral palsy (UCP). | Australia | Experimental pre-post, no control group | Pediatric | OT  Pre: 9  12m: 9 | Clinical practice guidelines for UL therapy for children with UCP. |
| Schreiber J, Marchetti G, Racicot B, Kaminski E.  2015 | The use of a knowledge translation program to increase use of standardized outcome measures in an outpatient pediatric physical therapy clinic: administrative case report | The purpose of this case report is to describe the use of a KT program to improve the knowledge and frequency of use of standardized outcome measures by pediatric physical therapists practicing in an outpatient clinic. | USA | Experimental pre-post, no control group | Pediatric | PT  Pre: 18 8m: 17 | Standardized pediatric outcome measures to provide patient outcome data to support frequency and duration recommendations. |
| Staines A, Amherdt I, Lécureux E, Petignat C, Eggimann P, Schwab M, Pittet D.  2017 | Hand Hygiene Improvement and Sustainability: Assessing a Breakthrough Collaborative in Western Switzerland | To assess hand hygiene improvement and sustainability associated with a Breakthrough Collaborative. | Switzerland | Experimental pre-post, no control group | Acute hospital | PT; Nurses; Physicians; Others  Pre: 4838 6 6m: unclear 12m: unclear | Hand hygiene compliance. |
| Stevenson K, Lewis M, Hay E.  2006 | Does physiotherapy management of low back pain change as a result of an evidence-based educational programme? | The aim of this study was to investigate if physiotherapists’ clinical management of patients with low back pain would change following an evidence-based education package, which utilized local opinion leaders and delivered the best evidence. | UK | Experimental pre-post with control group | Primary health care | PT  IG Pre: 17 PTs, 115 questionnaires.  6m: 16 PTs, 113 questionnaires;  CG Pre: 14 PTs, 35 questionnaires.  6m: 11PTs, 43 questionnaires. | Evidence-based practice of lumbar pain management. |
| Tilson J, Mickan S, Howard R, Sum J, Zibell M, Cleary L, Mody B, Michener L.  2016 | Promoting physical therapists' use of research evidence to inform clinical practice: part 3--long term feasibility assessment of the PEAK program | The purposes of this manuscript are to report 1) long-term outcomes regarding therapists’ EBP-related attitudes, self-efficacy, knowledge and skills, and self-reported behaviors, and 2) therapists’ adherence to participant-generated, evidence-based behaviors in patient care | USA | Experimental pre-post, no control group | Primary health care | PT  Pre: 18  6m: 16 | Integration of research evidence into clinical decision-making. |
| Tilson J, Martinz C, MacDowell S, D’Silva L, Howard R, Roth H, Skop K, Dannenbaum E, Farrell L.  2022 | Use of the knowledge to action model improved physical therapist adherence to a common clinical practice guideline across multiple settings: a multisite case series | The purpose of this study was to evaluate the impact of using a common process model to implement a single clinical practice guideline across multiple physical therapy clinical settings. | USA | Experimental pre-post with control group | Rehabilitation hospital | PT  Pre: 43 6m: 33 | Guidelines for peripheral vestibular hypofunction. |
| Vratsistas-Curto A, McCluskey A, Schurr K.  2017 | Use of audit, feedback and education increased guideline implementation in a multidisciplinary stroke unit | To increase the proportion of patients with stroke receiving best practice screening, assessment and treatment. | Australia | Experimental pre-post, no control group | Rehabilitation hospital | PT, OT, Nurses; Physicians; Others  Pre: 31  24m: unclear | Australian guideline recommendations for stroke rehabilitation. |
| Willett G, Johnson G, Jones K.  2011 | The effect of a hybrid continuing education course on outpatient physical therapy for individuals with low back pain | The purpose of this study was to evaluate the effect of a hybrid CE course on physical therapist use of interventions for individuals with acute LBP. | USA | Experimental pre-post, no control group | Other: Outpatient orthopaedic setting | PT  Pre: 43, 6m: 36 | Evidence-based practice of spinal manipulation for individuals with LBP. |
