# Supplementary material for: Sustainable implementation efforts in physio- and occupational therapy: a scoping review
Source: Implement Sci Commun. 2024 Dec 12;5:138. doi: 10.1186/s43058-024-00676-8 (PMC11636039; doi:10.1186/s43058-024-00676-8)
Supplement: Supplementary file 4 — Supplementary Material 4. [file 43058_2024_676_MOESM4_ESM.docx]

Additional file 4. Data extraction – implementation support and evaluation

| **Author** | **Implementation support strategy** | **Follow-up time** | **Sustainability support strategy** | **Theoretical basis** | **Outcome measure** | **Outcomes** |
| --- | --- | --- | --- | --- | --- | --- |
| *Auld M, Johnston L. | A 12-month multi-faceted Knowledge Translation intervention to target the identified barriers. 3-hour educational session that included information presented in written format, lecture format, physical demonstration and the opportunity to practice, and received personal feedback on their performance. Equipment to carry out the shortened Touch-in-10 assessment, written material on the basis for tactile assessment and detailed assessment forms providing the exact methodology for carrying out and scoring the assessments. Links to instructional videos indicating the exact methodology for performing a tactile assessment on a child. Additional one-on-one support through a mentoring program with the first author, to identify and plan tactile assessments within their current caseload. Regular contact to update them with information about tactile assessment and remind them of current research. Key opinion leaders, the senior professional team for both physiotherapists and occupational therapists, were identified and tasked with encouraging therapists to engage with the information and skills they were provided with during the training. Between the 3 and 12month assessment points another face-to-face meeting was carried out with the therapists to discuss any questions or concerns regarding tactile assessment. Throughout the timeframe of the intervention, ongoing mentoring support was offered. Online access to recent research and knowledge on tactile assessment, access to videos demonstrating each of the tactile assessment items. | 12 months | Ongoing mentoring support was offered to participating therapists. In addition, online  access to recent research and knowledge on tactile assessment, as well as access to videos demonstrating each of the tactile assessment items, were available to participating staff or new staff on a permanent basis. | Knowledge to Action Framework    The Theoretical Domains Framework    The Behavior Change Wheel | Questionnaire | Pre:  No tactile assessments: 5/12  Tactile assessments in less than 25% of the children: 6/12    Follow-up:  No tactile assessments: 1/8  Tactile assessments in less than 25% of the children: 3/8 |
| *Barton C, Kemp J, Roos E, Skou S, Dundules K, Pazzinatto M, Francis M, Lanning N, Wallis J, Crossley K. | 2-day training course: pre- and  post-workshop surveys evaluating knowledge and learning needs, lectures regarding osteoarthritis management, patient education and exercise-therapy skills training sessions, training to contribute patient outcomes to the national registry, and discussions about over-coming barriers to implementation. An implementation manual, ready-to-use patient education  materials (PowerPoint presentations and printable booklets), and access to online implementation resources (e.g. flyers, letter templates for referring doctors). | 12 months | No | The RE-AIM QuEST | Survey | Pre:  Discuss treatment goals: Most of the time: 40%,  All the time: 48%.  Prescribe neuromuscular exercise: Most of the time: 32%,  All the time: 22%.  Refer to, or provide supervised exercise programs: Most of the time: 34%,  All the time: 19%.  Discuss the importance of weight management: Most of the time: 34%, All the time: 22%.    Follow-up:  Discuss treatment goals: Most of the time: 31%,  All the time: 67%.  Prescribe neuromuscular exercise: Most of the time: 19%,  All the time: 76%.  Refer to, or provide supervised exercise programs: Most of the time: 39%,  All the time: 52%.  Discuss the importance of weight management: Most of the time: 36%, All the time: 52%.    79% (116/147) of physiotherapists who  responded to the 12-month survey had implemented GLA:D® |
| **Carlfjord S, Landén Ludvigsson M, Peolsson A, Peterson G. | Workshop 8h. Theoretical part: information about neck anatomy, neuromuscular functioning, symptoms associated with neck dysfunction, examination, and treatment based on recent research evidence. Practical part: analyzing movement patterns, focusing on deep neck muscle function, neck muscle endurance, and dizziness. A specific focus on the skill of differentiating cervical headache or dizziness from other kinds of headache/dizziness. Neck-specific exercise was practiced under the supervision of the teachers. | 12 months | No | No | Study-specific questionnaire: frequency of using specific diagnostic measure. | P-value at baseline, 3 months and 12 months:    a) Frequency of using various assessments for general neck pain with or without dizziness: p=0.08/0.77/0.07    b) Frequency of applying different treatment methods for patients with chronic WAD: p=0.21/0.04/0.25    c) Frequency of applying different treatment methods for patients with chronic WAD: p=0.01/0.01/0.02    d) Frequency of applying different treatment methods for patients with neck-related dizziness: p=0.61/0.47/0.67/0.54    e) Frequency of applying different treatment methods for patients with neck-related headache:  p=0.92/0.80/0.09 |
| **Fritz J, Wallin L, Söderlund A, Almqvist L, Sandborgh M | Implementation support period: 6 months. 10 outreach visits, peer coaching, educa-  tional materials, video recordings, individual goal setting, self-moni-  toring, manager support and access to written patient information | 12 months | The final meeting focused on sharing experiences related to problem-solving solutions and strategies for maintaining the behavioral medicine approach. | Social Cognitive Theory    PARIHS framework | Observed, self-reported and documented clinical behavior. An observation protocol  and a checklist were used | Observed clinical behavior: no sustained changes at 6 or 12 m.  Self-reported clinical behavior: no sustained changes at 6 or 12 m.  Documented clinical behaviour: no sustained changes at 6 or 12 m. |
| **Gross D, Lowe A. | Development and dissemination of a best practice resource guide and ‘tool kit’ for work disability prevention.  Creation of a network of peer-selected educationally influential clinicians.  Province-wide seminars to introduce the resources to practicing clinicians.  Use of the resources in the academic training curriculum. | 12 months | No | No | Survey | Few differences were observed in reported practice behaviors between the two survey periods. Contrary to the recommendations in the KTE intervention, more clinicians in the follow-up sample reported rarely discussing cases with physicians or insurance case managers, and these differences were  statistically significant. |
| *Kafri M, Levron Y, Atun-Einy O. | An interactive 20-hour course composed of an introductory presentation of t general concepts, practice variables, and learning strategies. The content was delivered using multiple educational strategies. Introduction of an illustrated conceptual model of motor learning elements, introduction to and a hands-on practice with a clinical self-directed form, each participant was given the opportunity to present his experience and a discussion was made about the challenges and appropriate strategies to overcome the implementation barriers. Participants received handouts of the presentations, a list of selected references and links to relevant educational resources. A structured form was used to guide the clinical thinking, planning, and clinical decision-making required for the intervention. This form outlines the practical steps required to implement the conceptual model. | 24 months | No | No | Physical therapists’ perceptions of Motor Learning (PTP-ML) questionnaire (reported implementation subscale) | Pre: Mean (SD): 3.02 (0.50)    Post: Mean (SD): 3.48 (0.42)    Follow-Up: Mean (SD): 3.59 (0.41) |
| **Karas S, Westerheide A, Daniel L. | Brief introduction to the techniques, the supporting evidence, demonstrations of the techniques, hands-on assessment of the PTs’ execution of the techniques, time to practice, and follow up questions. Following the 6 months programme, the PTs were given descriptions of the techniques, accompanied by pictures. | 6 months | Two and four months after the initial in-service, the therapists received an email-based research notes in a summarized fashion, with the purpose of  eliminating the barriers of locating, reading and applying the entire content of a research article. | No | Survey   Documented use of manual therapy during 6 months. | Pre:  Utilization: mean (SD): 3.77 (1.24)    Use om thoracic mobilization: 19.2%  Use om thoracic manipulation: 9.9%  Both: 7.5%    Follow-up:  Utilization: mean (SD): 4.08 (0.86)  No sign difference.    Use om thoracic mobilization: 42.3%  Use om thoracic manipulation: 14.8%  Both: 15.7% |
| *Lineker S, Bell M, Badley E.  2011 | Accredited inter-professional workshop and 6 months of activities to reinforce the learning: credible peer models (local multidisciplinary arthritis specialists), skill development, demonstration and feedback, goal setting, enhancing self-efficacy, addressing barriers, and providing reinforce-  ment. | 6 months | [The 6 months post-workshop included educational materials for staff and patients, posters, referral templates, community resource lists, and follow up of personal goals established at the workshops. Providers could also request additional training and resources.](http://www.arthritis.ca/gettingagrip) | Social Cognitive Theory | The Arthritis Community Research and Evaluation Unit (ACREU) Primary Care Survey | Significant  improvements in total best practices scores at 6 months post-workshop, with nurse practitioners and rehabilitation therapists improving the most (p < 0.05). |
| **McCluskey A, Lovarini M.  2005 | 2-day workshop combined with outreach support for eight months.  The workshop included lectures, practical sessions and small group discussion focused around six topics: the process of evidence-based practice; writing focused clinical questions; searching electronic databases; critical appraisal of qualitative and quantitative research; interpreting statistics in randomised controlled trials; and overcoming barriers/making the change to evidence-based practice.   Support involved email and telephone contact and a workplace visit. | 8 months | E-mail and  telephone contact and an optional workplace visit. Participants were invited to develop a learning contract. An email list was set up to facilitate commu-  nication. Information distributed via this list included  resources and websites and answers to frequently asked  questions. Reminders and individual feedback were pro-  vided about the assignment. | Social Cognitive Theory    Diffusion and innovations    Transtheoretical model    Adult learning theories | Activity diary (% engaging/not engaging in search and appraisal activities) and assignment completion. | The only statistically significant difference was a decrease  – not an increase as hypothesised – in the proportion of therapists engaged in searching between Time 1 and Time 5. |
| *McDonnell B, Stillwell S, Hart S, Davis R.  2018 | Implementation support period: 2 years. Laminated pocket cards for easy reference  throughout patient care.  Interactive educational sessions: review  of the benefits of using STOM as part  of regular clinical practice, as well as  the presentation of the work completed  by the Measures Selection Committee.  Key points regarding proper  administration, psychometric properties  relevant to clinical decision making  in acute care, and considerations  for application of STOM to specific  patients. Case Conference and Journal Club. Follow-up discussion and feedback from staff was encouraged at monthly staff meetings.  Opinion leaders: to address barriers such as lack of familiarity with STOM and lack of training in administration of STOM. Opinion leaders also promoted facilitators such as support of colleagues in the use of STOM.  Audit of medical records to assess the frequency of STOM use. Based on the results of this audit, potential areas for improvement were identified and all physical therapists were provided with feedback regarding their frequency of using STOM. | 24 months | Follow-up discussion and feedback from staff was encouraged at monthly staff meetings. | No | Medical chart review | Statistically significant increase in the use (primary outcome)  of STOM was observed following the implementation of  KT strategies. |
| *Meerhoff F, van Dulmen S, Maas M, Heijblom K, Nijhuis-van der Sanden M, Van der Wees P.  2017 | Implementation support period: 15-18 months. Opinion leaders, audit and feedback with peer assessment, educational outreach, educational meetings, and 5 workshops. | 18 months | No | The Implementation Model (Grol & Wensing) | Medical chart review and PROM use self-assessment questionnaire. | PTs who reported using PROMs with the majority of their patients increased significantly to 10.7% (95% CI 3.8–17.6; P-value ≤.001).    Actual PROM use  increased from 25.5% to 71.2%, and the  overall pre- and posttreatment PROM  use increased from 12.2% to 39.5%. |
| *Moore J, Carpenter J, Doyle A, Doyle L, Hansen P, Hahn B, Hornby TJ, Roth H, Spoeri S, Tappan R, Van Der Laan K.  2018 | Typically, 4 champion education sessions  are held each year per discipline: providing educational sessions, working with stakeholders to overcome site-specific barriers, modeling and acting as a local mentor, and providing feedback about implementation. | 6 years | Incentives, leadership infrastructure, and consistent exposure to the project. | Knowledge to Action Framework | Survey | Substantial increase in use of outcome measures  after 3 years (74%) and 6 years (91%) and evidence-based interventions after 3 years (62%) and 6 years (82%). After 3 years significant differences (P< .01) in effect of the Battery of Rehabilitation Assessments and Interventions on practice were identified between therapists who were directly involved in the  project and Interventions compared with uninvolved therapists. After 6 years, no significant differences existed between involved and uninvolved therapists. |
| *Moore J, Bø E, Erichsen A, Rosseland I, Halvorsen J, Bratlie H, Hornby G, Nordvik JE.  2021 | Implementation support period: about 2 years. 2-day KTA workshop, 1-day course on evidence.  Online and in-person training.  Educational interventions, accessing funding, changing physical structure and equipment, promoting adaptability of HIT,  conducting local consensus discussions, and others | 24 months | Weekly meetings to ensure that HIT was a high priority, discuss the inclusion of patients in the program, and provide HIT mentoring. The clinicians participated in monthly professional meetings to review HIT articles and provide feedback on clinicians’ outcome measurements and treatment characteristics. The clinicians revised the stroke treatment guideline to include HIT at the 2 sites and created a HIT training plan for new employees. | Knowledge to Action Framework    The CFIR | Questionnaire | Significant change in 9 out of 10 questions. |
| *Moore J, Virva R, Henderson C, Lenca L, Butzer J, Lovell L, Roth E, Graham I, Hornby G.  2022 | Implementation support period: 14 months. Co-developing and executing the plan with clinicians and leaders, implementation facilitation, implementation leadership, and a bundle of knowledge translation interventions that targeted barriers.  Educational interventions, leadership support, process changes, audit and feedback, purchasing of equipment, and environmental modifications. | 48 months | No | Knowledge to Action Framework    The Theoretical Domains Framework | Medical chart review and survey | Pre:  Adherence: 46%    Follow-up:  Adherence: 6 months: 85%, 48 months: 95.2%. Use of the 10MWT significantly improved between 2015, 2016, and 2018 (2015: median, 3.0; range, 2.0-4.0; 2016: median, 5.0; range, 4.0-5.0; 2018: median, 5.0; range, 4.25-5.0; P=.006). Post hoc testing indicated differences between 2015 and 2018 (P=.03). |
| *Moseng T, Dagfinrud H, Østerås N.  2019 | 1 day inter-active workshop. General update on OA including epidemiology, clinical features and treatment recommendations:  -Education in delivery of a standardised patient-education program  -Education and practical training in delivery of an individually tailored semi-standardised exercise intervention and use of performance tests  -Education about healthy nutrition and weight management   The multidisciplinary workshop lasted 1.5 h and included a general update on OA including treatment recommendations and a discussion on OA care. The PTs received access to the “ready-to-use” standardized patient education program (Power-Point file and manuscript). They also received access to a database with recommended exercises and dose. | 6 months | No | Framework of Carroll & co | Patient reported data: yes/no questions. | PE: IG: 199 (70%), CG: 6 (5%), 95% CI: (17.0, 95.1), p<0.001    IN: IG: 190 (67%), CG: 51 (47%), 95% CI: (1.5, 3.6), p<0.001    RE: IG: 209 (74%), CG: 45 (41%), 95% CI: (2.5, 6.3), p<0.001    CE: IG: 182 (64%), CG: 34 (31%), 95% CI: (2.4, 6.3), p<0.001    PT: IG: 1(0.4%), CG: 8 (7%), 95% CI: (0.01, 0.4), p<0.001    WR: IG: 33 (25%), CG: 5 (9%), 95% CI: 5 (1.3, 9.4), p<0.001 |
| *Novak I, McIntyre S.  2010 | 1-day EBP workshop using competency-based learning followed by individual coaching. Real clinical examples from the medical records of patients seen by the participants. Rehearsal and confidence building at question construction, searching and appraisal, using supported self-directed learning activities. Role-modelling from seniors, via formal presentations of a successfully completed ‘critically appraised topics’ (CATs), production of a CAT in small groups using a peer-support learning model and development of a shared action plan about future EBP implementation priorities within the organization.  Workplace supports:  an overall change process, which facilitated systematic and incremental implementation of EBP;  strategic planning for identifying EBP priorities and allocating the necessary resources to support implementation; development of management and supervision structures to provide the necessary support; show-casing clinical leadership that role-modelled exemplar evidence-based clinical practice, provision of peer support; working through the barriers to change and provide strategic support to the change process; and development of working groups that used a peer support model to maintain individual motivation and to provide practical support to staff during the learning phase | 18 months | No | Tropman’s organizational rubrik | Frequency counting of role description performance indicators, including number of CATs conducted about core practice and disseminated on the staff intranet, and number of peer-reviewed conference presentations given by participants using an EBP methodology rather than an expert opinion mode. | Pre:  1) 0.11  2) 3%    Follow-up:  1) 0.27  t=5.55, p< 0.001, 95% CI: 0.17–0.35    2) 97%  t= 24.39, p< 0.001, 95% CI: 0.86–1.03 |
| **Olsen N, Bradley P, Espehaug B, Wammen Nortvedt M, Lygren H, Frisk B, Bjordal JM.  2015 | A multifaceted and clinically integrated training program (6 ECTS-credits), delivered to over a six-month period: workshops, assignments, supervision, and exams. The workshops were a mixture of lectures and small-group activities that required participants to be interactive. Four half-day workshop sessions covering the EBP steps and processes was delivered equentially over a six-week period. Between and after workshops, five individual written assignments (Week 2, 4, 7, 1, 21) were required from the participants. To ensure clinically integrated learning of EBP, each assignment required participants to reflect on and describe how to apply the EBP steps in real clinical situations. The EBP tool is a learning tool intended to provide health care professionals with practical EBP skills. The EBP steps and processes registered in one document facilitate the learning process and the possibility of receiving and giving feedback. For each assignment, participants received supervision via phone and/or email, in addition to guidance from a librarian when necessary. The final exam took form of an individual oral presentation, where participants focused on how to apply the EBP steps to a real patient situation and how to supervise students in the EBP process (Week 27). The exam was assessed as pass/fail. | 6 months | No | Experiential learning theory | Questionnaire: the EBP Implementation Scale | Pre:  IG: 7.7, CG: 8.9  MD between groups: -1.1  95% CI: (-5-2.8), p: 0.570    Follow-up:  IG: 12.3, CG: 10.5  MD between groups: 1.8  95% CI: (-4.5-8.1), p: 0.574    MD within groups  IG: 4.6 (1.7-7.5), p: 0.002  CG: 1.7, (-4.2-7.5), p: 0.574 |
| **Pöder U, Fogelberg-Dahm M, Wadensten B.  2011 | Training staff both in care and in the technical aspects of using the EB-SCP in the EHR and was carried out by one of the authors during the first weeks. Thereafter, the head nurses and the nurses responsible for nursing documentation on the wards were responsible for the on-going work and introduction to new staff. | 12 months | No | No | Questionnaire | Pre: Use of an individual care plan: 27 (79% of n= 34)    Follow-up: Use of an individual care plan: 34 (94% of n= 37). No difference. |
| *Romney W, Salbach N, Parrott JS, Deutsch J.  2020 | Intervention group:  4 x 1-h lunch meetings during 2 months The KB applied the following strategies: educational outreach visits, handouts (with instructions on how to administer and interpret the test), a binder with articles, documentation changes, goal setting, audit and feedback, environmental changes, organizational support and facilitation, and PTs’ engagement and social support. Instructions of how to calculate gait speed was added to all examination packets. Audit and feedback were provided twice by the KB at months 2 and 9. The KB enlisted the support of the supervisor to help with scheduling meetings, intervention design, documentation changes, and goal setting.   Control group:  Trained individually by the supervisor between months 4 and 8 of the study. Training included a review of the protocol of the 4MWT and psychometrics of the test. The PTs were given the educational material that was provided by the KB and trained on how to document in the patient charts. Training was approximately 15 minutes. | 8 months | No | Knowledge to Action Framework    The Theoretical Domains Framework | Medical chart review | Documented use at the 3 additional time points was not significantly different for initial evaluation (0-2 to 2-4 months [mean = 34%, SD = 38%, P= .21], 2-4 to 4-6 months [mean = 57%, SD = 34%, P= .99], and 4-6 to 6-8 months [mean = 63%, SD = 21%, P= .99]) and discharge (0-2 to 2-4 months [mean = 53%, SD = 39%, P= .99], 2-4 to 4-6 months [mean = 52%, SD = 25%, P= .99], and 4-6 to 6-8 months [mean = 59%, SD = 32%, P= .99]).    The comparison group had increased documentation of gait speed at 4 to 6 months (initial evaluation 24% and discharge 35%) and 6 to 8 months (initial evaluation 25% and discharge 47%) |
| *Romney W, Wormley M, Veneri D, Oberlander A, Grevelding P, Rice J, Moore J.  2022 | Implementation support period: 24 months. Use of opinion leaders, educational in-service, documentation changes in the electronic medical record and paper examination templates, environmental changes to serve as reminders, organizational and therapy administrative support, goal setting, social engagement, and audit and feedback. | 24 months | Chart audits and verbal feedback about documentation adherence goals which remained as part of the monthly staff meetings. Open discussions about barriers and new KT strategies were reviewed when adherence was less than desired. | Knowledge to Action Framework | Manual chart review and an automated report from the electronic medical record. | Pre: Range of use: 53-94%    Follow-up:  OR 12-18: 8.5, 95%CI 6.0–12.1 (p < .001) OR 18-24: 10.8, 95%CI 7.6–15.3. (p < .001) |
| *Russell D, Rivard L, Walter S, Rosenbaum P, Roxborough L, Cameron D, Darrah J, Bartlett D, Hanna S, Avery L.  2010 | Implementation support period: 6 months. Self-learning, needs assessments, presentations, group discussions, accessing and modifying resources, one-on-one interactions with various stakeholders, networking with other KBs, accessing computer support, and collaborative measurement and scoring of clients. | 18 months | No | Knowledge to Action Framework | Questionnaire about use of the measurement tools | Pre-post: OR, 95% CI, p  GMFCS: 18.2, 5.5-60.1, <0.01; 11.8, 2.4-57.7, <0.01  GMFM-88: 2.7, 0.9-7.8, 0.07  GMFM-66: 6.2, 2.2-17.6, <0.01  MGC: 3.3, 1.1-9.8, 0.03    6-12m: OR, 95% CI, p  GMFCS: 1.8, 0.3-10.6, 0.49  GMFM-88: 1.2, 0.4-3.3, 0.73  GMFM-66: 0.9, 0.2-4.2, 0.87  MGC: 0.9, 0.2-4.3, 0.87    12-18m: OR, 95% CI, p  GMFCS: 0.8, 0.1-4.8, 0.80  GMFM-88: 1.2, 0.4-3.8, 0.77  GMFM-66: 1.7, 0.3-8.8, 0.53  MGC: 0.4, 0.1-1.1, 0.07 |
| *Sakzewski L, Ziviani J, Boyd R  2016 | 2 half day workshops and 1 day workshop. Audit and feed-back, barrier identification, interactive education and training targeting identified barriers. Audit and feedback and barrier identification occurred concurrently and results informed content of the education/training component of the intervention. | 12 months | No | Control Theory | Medical chart review | 12 criteria: Range of % change: +8 to +38, OR range 1.4-10.8 |
| *Schreiber J, Marchetti G, Racicot B, Kaminski E.  2015 | 2-hour practice-based workshop: presession reading materials, brief lectures on content, opportunities to practice standardized testing procedures, and identification of site-specific barriers and suggested solutions. Hardcopy binder with all workshop materials, including test score sheets, normative values where available, and instructions for implementing and interpreting each standardized outcome measure. A decision-making algorithm. A team website, along with video demonstrations for the outcome measures. Team discussion board with required postings by all staff. 1-hour follow-up sessions approximately 2 months after the first workshop: follow-up staff meetings and e-mail communication about implementation of workshop information.  Local opinion leaders. | 8 months | No | Knowledge to Action Framework | Data from the electronic medical record query.    The Self-Assessment of Knowledge and Frequency of Performance (SAKFP). | Pre:  PEDI: 0, GMFM-66: 0, GMFM-88: 6, TUG: 12, TUDS: 1, 30-s walk test: 0.    Follow-up:  PEDI: 82, GMFM-66: 8, GMFM-88: 2, TUG: 24, TUDS: 70, 30-s walk test: 32.    SAKFP: Selection: mean change: 11.6 +/- 5.9 points, P<.001 Administration: P<.001 Interpretation: mean change: 4.2 +/- 4.6 points, P<.006 Sharing information: mean change: 2.1 +/- 4.6 points, P<.091 |
| *Staines A, Amherdt I, Lécureux E, Petignat C, Eggimann P, Schwab M, Pittet D.  2017 | A project team at each hospital attend a 3-days learning session.  6 learning session in 18 months: system change, training, and education, performance monitoring and feedback, workplace reminder, leadership and institutional safety climate. | 12 months | No | No | Open and direct observations. | Pre: Compliance: 61.9%    Compliance 6 m: 88.3% (p<.001)    Compliance 12 m: 88.9% (p=.248 6 to 12 months) |
| **Stevenson K, Lewis M, Hay E.  2006 | Intervention group:  Interactive evidence-based educational programme (teaching, discussion, reflective thinking, active experimentation, peer group teaching).  Opinion leaders.  2.5h critical appraisal and literature searching skills and 2.5h on the latest management for patients with acute and chronic low back pain.   Control group: a standard in-service training package on clinical management of knee dysfunction and pathology. | 6 months | No | No | A standardized ‘discharge summary’ questionnaire | A little change in reported use of therapies (time spent and importance). CG: more likely to use ‘acupuncture’, ‘encourage to undertake activities themselves’ and give ‘postural advice’. IG: more likely to give ‘advice to increase activity level’ and ‘change attitudes/beliefs about pain’ but less likely to ‘encourage to undertake activities them-selves’. |
| *Tilson J, Mickan S, Howard R, Sum J, Zibell M, Cleary L, Mody B, Michener L.  2016 | Acquiring managerial leadership support and electronic resources. A 2-day learner-centered EBP training workshop.  5 months of guided small group work synthesizing research evidence into a locally relevant list of actionable, evidence-based clinical behaviors for therapists treating persons with musculoskeletal lumbar conditions–the Best Practices List. Review and revision of the Best Practices List, culminating in participant agreement to implement the behaviors in practice. | 6 months | No | Knowledge to Action Framework    Social Cognitive Theory    Adult learning theories | EBP Implementation Scale.  Medical chart review. | Pre: Self-reported behavior (mean, SD): 25.5 (8.5)    Follow-up:  Self-reported behavior (mean, SD): 30.3 (8.2), p=0.999 (post-6m)    Chart reviews: Only 1 of 38 had a statistically significant change |
| *Tilson J, Martinz C, MacDowell S, D’Silva L, Howard R, Roth H, Skop K, Dannenbaum E, Farrell L.  2022 | Implementation support period: 6 months. Monthly audit and feedback meetings, communities of practice, educational materials, educational meetings, local consensus process, local opinion leaders, reminders, resources provided to therapists to offer to patient. | 6 months | Each site determined a plan to continue to promote adherence to the target therapist behaviors after the six-month intervention. | Knowledge to Action Framework | Medical chart review, tally counts, survey. | Adherence to target behaviors was mixed. Among four sites with similar target behaviors, three had multiple areas of statistically significantly improved adherence and one site had limited improvement. Success was most common with behaviors related to documentation and offering patients low technology resources to support home exercise. |
| *Vratsistas-Curto A, McCluskey A, Schurr K.  2017 | Implementation support period: 10 months and 7 months. Four cycles of audit and feedback, identifying determinants of practice, provision of educational materials, group education sessions to assist staff with change. | 24 months | No | The Theoretical Domains Framework    The Behavior Change Wheel    COM-B | Medical chart review. | Pre:  PT: Treadmill training: 18% (95% CI −5% to 41%) Sitting balance training: 25% (95% CI −5% to 55%)  OT: Standardised assessment of sensation: 0%, Provision of sensory training: 0%    Follow-up:  PT: Treadmill training: 50% (95% CI −19% to 119%) Sitting balance training: 67% (95% CI 29% to 104%) OT: Standardised assessment of sensation: 0%, Provision of sensory training: 100% |
| *Willett G, Johnson G, Jones K.  2011 | Online educational intervention within a transitional doctor of physical therapy program: reading three journal articles concerning spinal manipulation for LBP patients, completing multiple choice and short answer examination questions for each article. 1 hour lecture that summarized the current evidence, 1 hour laboratory experience consisting of a review and practice session of the lumbosacral manipulation technique used in the study. During lab, the instructors demonstrated a specific spinal manipulation intervention which was followed by student practice with multiple partners. Each subject was required to demonstrate the skills and receive feedback from an instructor during the session. | 6 months | No | Kirkpatrick’s taxonomy of training criteria | Survey | Pre:  Utilization of intervention: 21%    Preferred intervention: 3%    Follow-up:  Utilization of intervention: 69%    Preferred intervention: 11%, p=0.005 |
