# Supplementary material for: Sustainable implementation efforts in physio- and occupational therapy: a scoping review
Source: Implement Sci Commun. 2024 Dec 12;5:138. doi: 10.1186/s43058-024-00676-8 (PMC11636039; doi:10.1186/s43058-024-00676-8)
Supplement: Supplementary file 5 — Supplementary Material 5. [file 43058_2024_676_MOESM5_ESM.docx]

Additional file 5. Implementation support strategies.

| **Study** | **Frequency** | **Implementation support strategy** |
| --- | --- | --- |
| Auld & Johnston, 2019* | 10 | Educational materials: written information  Educational meetings: lecture  Physical demonstration/video  Training/practice  Personal feedback  Mentoring/one-to-one support  Opinion leaders  Problem solving  Resources: scoring sheets, record forms  Reminders: regular contact, encouragement |
| Barton et al, 2021* | 5 | Educational meetings: lecture  Training/practice  Problem solving  Educational materials: ppt, booklets, written information  Resources: online flyers, templates |
| Carlfjord et al, 2021** | 2 | Educational meetings: lecture  Training/practice |
| Fritz et al, 2020** | 10 | Supervising, Outreach visits  Communities of practice: peer coaching  Educational materials  Educational meetings: lecture  Modeling  Video recordings (audit)  Goal setting  Self-monitoring  Leadership support  Resources: written patient information |
| Gross & Lowe, 2009** | 5 | Educational materials: supporting guide  Resources: outcome measures, vignettes  Network of peer-selected educationally influential clinicians  Demonstration  Educational meetings: seminars |
| Kafri et al, 2023* | 7 | Educational meetings: instructions  Educational materials  Training/practice  Demonstrations  Audit and feedback  Problem solving  Resources |
| Karas et al, 2016** | 5 | Educational meetings: lecture  Demonstration  Training/practice  Educational material: written information  Resources: e-mail with articles |
| Lineker et al, 2011* | 8 | Educational meetings: workshops  Review of examination with trained patient educators  Training/practice  Communities of practice: small group discussions  Action plans  Goal setting  Educational materials  Resources: posters, templates |
| Mc Cluskey & Lovarini, 2005** | 8 | Educational meetings: workshop, lecture, outreach visits  Training/practice  Communities of practice: small-group discussion  Problem solving  Individual goals  Mentoring: e-mail, telephone contact, optional workplace visit  Resources: website, FAQ  Audit and feedback |
| McDonnell et al, 2018* | 5 | Communities of practice: case conferences, journal club  Written materials: laminated pocket cards  Opinion leader  Mentoring  Audit and feedback |
| Meerhoff et al, 2017* | 4 | Opinion leader  Audit and feedback  Communities of practice: peer assessment  Educational meetings: outreach, workshops |
| Moore et al, 2018* | 7 | Educational meetings  Problem solving  Modeling  Mentoring  Feedback  Local opinion leader/champions  Support to champions |
| Moore et al, 2021* | 9 | Educational meetings: workshop  Resources: manual, forms  Problem solving  Patient demonstration  Local consensus discussion  Online and in-person training  Environmental modifications: changing physical structure  Audit and feedback  Reminders: monthly newsletter |
| Moore et al, 2022* | 10 | Educational meetings  Goal setting  Leadership support  Audit and feedback  Resources: equipment, research assistant  Reminders: e-mails  Rewards  Communities of practice: team conferences  Environmental modifications: incorporated in medical records  Organizational support (Process changes: integration into organizational goals and vision) |
| Moseng et al, 2019* | 3 | Educational meetings: workshop, lecture  Training/practice  Resources: ppt, manuscript, access to database |
| Novak & McIntyre, 2010* | 6 | Educational meetings: workshop  Individual coaching  Demonstration/Modeling  Communities of practice: peer-support  Action plan  Problem solving |
| Olsen et al, 2015** | 5 | Educational meetings: workshop, lecture  Communities of practice: small-group discussion  Supervision  Written assignment  Educational material: the EBP tool |
| Pöder et al, 2011** | 2 | Local opinion leader  Training/practice |
| Romney et al, 2020* | 8 | Educational meetings: outreach  Educational materials: articles, handouts  Goal setting  Audit and feedback  Environmental changes  Organizational support  Knowledge broker  Local consensus discussion |
| Romney et al, 2022* | 12 | Local opinion leaders  Reminders: environmental changes  Educational in-service  Demonstrations  Training/practice  Organizational support and administration support  Goal setting  Communities of practice: group discussions, peer assessment  Audit and feedback  Problem solving  Environmental modifications: changes in electronic medical record  Resources: paper examination templates |
| Russel et al, 2010* | 8 | Knowledge brokers/local opinion leaders  Educational meetings: lecture  Communities of practice: group discussion  One-on-one interactions with stakeholders  Peer-support/networking with other knowledge brokers  Resources: computer support  Collaborative practice sessions  Financial resources for knowledge brokers |
| Sakzewski et al, 2016* | 8 | Educational meetings: workshop  Training/practice  Demonstration (video case)  Problem solving  Resources: templates for home programs and home practice logs  Communities of practice: small group discussions  Audit and feedback  Environmental modifications |
| Schreiber et al, 2015* | 10 | Educational meetings: workshop, lecture  Educational materials  Training/practice  Problem solving  Demonstrations (video)  Local opinion leaders  Audit and feedback  Communities of practice: online discussion board with peers  Reminders: newsletter updates  Resources: test score sheets |
| Staines et al, 2017* | 7 | Training/practice  Educational meetings: lecture  Audit and feedback  Reminders  Self-monitoring  Demonstration (video)  Leadership and institutional safety climate |
| Stevenson et al, 2006** | 3 | Educational meetings  Training/practice  Local opinion leaders |
| Tilson et al, 2016* | 5 | Leadership support  Educational materials: electronic resources, instructions  Training/practice  Educational meetings: workshop  Communities of practice: guided small group work |
| Tilson et al, 2022* | 8 | Audit and feedback  Educational materials: guidelines  Educational meetings  Local opinion leaders  Reminders: digital reminders  Problem solving  Communities of practice: small group discussions  Resources provided to therapists to offer to patients |
| Vratsistas-Curto et al, 2017* | 6 | Audit and feedback  Educational materials  Educational meetings: instructions  Training/practice  Demonstrations  Environmental restructuring |
| Willett et al, 2011* | 5 | Educational materials: articles  Educational meetings: lecture  Training/practice  Demonstration  Audit and feedback |

*Reporting sustained results, **Reporting unsustained results
