# Supplementary material for: Sustainable implementation efforts in physio- and occupational therapy: a scoping review
Source: Implement Sci Commun. 2024 Dec 12;5:138. doi: 10.1186/s43058-024-00676-8 (PMC11636039; doi:10.1186/s43058-024-00676-8)
Supplement: Supplementary file 6 — Supplementary Material 6. [file 43058_2024_676_MOESM6_ESM.docx]

Additional file 6. Behavior change techniques [34] used to support the implementation. Bolded techniques are reported in the scientific paper.

| **Study** | **Frequency** | **BCT** |
| --- | --- | --- |
| **Auld & Johnston, 2019*** | 9 | 2.2 Feedback on behavior  **2.3** **Self-monitoring of behaviour**  **4.1 Instruction on how to perform the behavior**  **5.1 Information about health consequences**  6.1 Demonstration of the behavior  **7.1** **Prompts/cues**  **8.1 Behavioral practice/rehearsal**  **12.1 Restructuring the physical environment**  **15.1 Verbal persuasion about capability** |
| Barton et al, 2021* | 5 | 1.2 Problem solving  4.1 Instruction on how to perform the behavior  6.1 Demonstration of the behavior  8.1 Behavioral practice/rehearsal  12.5 Adding objects to the environment |
| Carlfjord et al, 2021** | 2 | 4.1 Instruction on how to perform the behavior  8.1 Behavioral practice/rehearsal |
| Fritz et al, 2020** | 10 | 1.2 Problem solving  1.3 Goal setting (outcome)  1.5 Action planning  2.2 Feedback on behavior  2.3 Self-monitoring of behaviour  3.1 Social support (unspecified)  4.1 Instruction on how to perform the behavior  6.1 Demonstration of the behavior  8.1 Behavioral practice/rehearsal  12.5 Adding objects to the environment |
| Gross & Lowe, 2009** | 2 | 3.1 Social support (unspecified)  4.1 Instruction on how to perform the behavior |
| Kafri et al, 2023* | 6 | 1.2 Problem solving  2.2 Feedback on behavior  4.1 Instruction on how to perform the behavior  6.1 Demonstration of the behavior  8.1 Behavioral practice/rehearsal  Adding objects to the environment |
| Karas et al, 2016** | 3 | 4.1 Instruction on how to perform the behavior  6.1 Demonstration of the behavior  8.1 Behavioral practice/rehearsal |
| Lineker et al, 2011* | 8 | 1.2 Problem solving  1.3 Goal setting (outcome)  1.4 Action planning  1.6 Discrepancy between current behavior and goal  2.2 Feedback on behavior  4.1 Instruction on how to perform the behavior  6.1 Demonstration of the behavior  8.1 Behavioral practice/rehearsal |
| Mc Cluskey & Lovarini, 2005** | 7 | 1.2 Problem solving  1.3 Goal setting (outcome)  2.2 Feedback on behavior  3.2 Social support (practical)  6.1 Demonstration of the behavior  7.1 Prompts/cues  8.1 Behavioral practice/rehearsal |
| McDonnell et al, 2018* | 4 | 1.2 Problem solving  2.2 Feedback on behavior  3.1 Social support (unspecified)  7.1 Prompts/cues |
| Meerhoff et al, 2017* | 3 | 2.2 Feedback on behavior  3.1 Social support (unspecified)  4.1 Instruction on how to perform the behavior |
| Moore et al, 2018* | 4 | 1.2 Problem solving  2.2 Feedback on behavior  3.2 Social support (unspecified)  6.1 Demonstration of the behavior |
| Moore et al, 2021* | 8 | 1.2 Problem solving  2.2 Feedback on behavior  3.1 Social support (unspecified)  4.1 Instruction on how to perform the behavior  6.1 Demonstration of the behavior  7.1 Prompts/cues  12.1 Restructuring the physical environment  12.5 Adding objects to the environment |
| Moore et al, 2022* | 6 | 1.2 Problem solving  2.2 Feedback on behavior  2.7 Feedback on outcome(s) of behavior  7.1 Prompts/cues  12.1 Restructuring the physical environment  12.5 Adding objects to the environment |
| Moseng et al, 2019* | 3 | 4.1 Instruction on how to perform the behavior  4.2 Information about antecedents  8.1 Behavioral practice/rehearsal |
| Novak & McIntyre, 2010* | 6 | 1.2 Problem solving  1.4 Action planning  3.1 Social support (unspecified)  4.1 Instruction on how to perform the behavior  6.1 Demonstration of the behavior  8.1 Behavioral practice/rehearsal |
| Olsen et al, 2015** | 4 | 1.2 Problem solving  2.2 Feedback on behavior  4.1 Instruction on how to perform the behavior  8.1 Behavioral practice/rehearsal |
| Pöder et al, 2011** | 2 | 4.1 Instruction on how to perform the behavior  8.1 Behavioral practice/rehearsal |
| Romney et al, 2020* | 7 | 1.3 Goal setting (outcome)  2.7 Feedback on outcome(s) of behavior  3.2 Social support (practical)  3.3 Social support (emotional)  4.1 Instruction on how to perform the behavior  8.1 Behavioral practice/rehearsal  12.1 Restructuring the physical environment |
| Romney et al, 2022* | 9 | 1.2 Problem solving  1.3 Goal setting (outcome)  2.7 Feedback on outcome(s) of behavior  3.1 Social support (unspecified)  4.1 Instruction on how to perform the behavior  6.1 Demonstration of the behavior  7.1 Prompts/cues  8.1 Behavioral practice/rehearsal  12.1 Restructuring the physical environment |
| Russel et al, 2010* | 4 | 1.2 Problem solving  2.3 Self-monitoring of behaviour  3.1 Social support (unspecified)  4.1 Instruction on how to perform the behavior |
| Sakzewski et al, 2016* | 5 | 1.2 Problem solving  4.1 Instruction on how to perform the behavior  6.1 Demonstration of the behavior  8.1 Behavioral practice/rehearsal  12.5 Adding objects to the environment |
| Schreiber et al, 2015* | 6 | 1.2 Problem solving  3.1 Social support (unspecified)  4.1 Instruction on how to perform the behavior  6.1 Demonstration of the behavior  8.1 Behavioral practice/rehearsal  12.5 Adding objects to the environment |
| Staines et al, 2017* | 8 | 1.3 Goal setting (outcome)  2.7 Feedback on outcome(s) of behavior  3.1 Social support (unspecified)  4.1 Instruction on how to perform the behavior  7.1 Prompts/cues  8.1 Behavioral practice/rehearsal  10.4 Social reward  12.1 Restructuring the physical environment |
| Stevenson et al, 2006** | 2 | 3.1 Social support (unspecified)  4.1 Instruction on how to perform the behavior |
| Tilson et al, 2016* | 4 | 3.1 Social support (unspecified)  4.1 Instruction on how to perform the behavior  8.1 Behavioral practice/rehearsal  12.1 Restructuring the physical environment |
| Tilson et al, 2022* | 6 | 1.2 Problem solving  2.7 Feedback on outcome(s) of behavior  3.1 Social support (unspecified)  4.1 Instruction on how to perform the behavior  7.1 Prompts/cues  12.5 Adding objects to the environment |
| **Vratsistas-Curto et al, 2017*** | 11 | **2.2** **Feedback on behavior**  2.3 Self-monitoring of behaviour  **4.1 Instruction on how to perform the behavior**  **5.1 Information about health consequences**  **6.1 Demonstration of the behavior**  **7.1 Prompts/cues**  **8.1 Behavioral practice/rehearsal**  **9.1 Credible source**  12.1 Restructuring the physical environment  **12.5 Adding objects to the environment**  **15.3 Focus on past success** |
| Willett et al, 2011* | 4 | 2.2 Feedback on behavior  4.1 Instruction on how to perform the behavior  6.1 Demonstration of the behavior  8.1 Behavioral practice/rehearsal |

*Reporting sustained results, **Reporting unsustained results
